# Highly differential count of circulating and tumor infiltrating immune cells in patients with non-HCV/non-HBV hepatocellular carcinoma

**DOI:** 10.1007/s00262-021-03061-9

**Published:** 2021-09-28

**Authors:** Markus Bo Schoenberg, Tong Zhu, Jingcheng Hao, Julian Nikolaus Bucher, Xiaokang Li, Xinyu Li, Yongsheng Han, Dionysios Koliogiannis, Michaela Svihla, Markus Otto Guba, Jens Werner, Alexandr V. Bazhin

**Affiliations:** 1 Department of General, Visceral, and Transplant Surgery, Ludwig-Maximilians-University Munich, Marchioninistraße 15, 81377 Munich, Germany; 2 German Cancer Consortium (DKTK), Partner Site Munich, Munich, Germany; 3 Transplantation Center Munich, Hospital of the LMU, Campus Grosshadern, Munich, Germany; 4 Bavarian Cancer Research Center (BZKF), Munich, Germany

**Keywords:** Hepatocellular carcinoma, Flow cytometry, Tumor immunology, Immunology, Liver resection

## Abstract

**Background:**

Liver transplantation and liver resection are curative options for early hepatocellular carcinoma (HCC). The outcome is in part depended on the immunological response to the malignancy. In this study, we aimed to identify immunological profiles of non-HCV/non-HBV HCC patients.

**Methods:**

Thirty-nine immune cell subsets were measured with multicolor flow cytometry. This immunophenotyping was performed in peripheral blood (PB) and tumor specimens of 10 HCC resection patients and 10 healthy donors. The signatures of the highly differential leukocyte count (hDIF) were analyzed using multidimensional techniques. Functional capability was measured using intracellular IFN-*γ* staining (Trial Registration DRKS00013567).

**Results:**

The hDIF showed activation (subsets of *T*-, *B*-, NK- and dendritic cells) and suppression (subsets of myeloid-derived suppressor cells and *T*- and *B*-regulatory cells) of the antitumor response. Principal component analysis of PB and tumor infiltrating leukocytes (TIL) illustrated an antitumor activating gradient. TILs showed functional capability by secreting IFN-*γ* but did not kill HCC cells.

**Conclusions:**

In conclusion, the measurement of the hDIF shows distinct differences in immune reactions against non-HBV/non-HCV HCC and illustrates an immunosuppressive gradient toward peripheral blood.

**Trial Registration:**

DRKS00013567

**Supplementary Information:**

The online version contains supplementary material available at 10.1007/s00262-021-03061-9.

## Introduction

Hepatocellular carcinoma (HCC) is the most common type of primary hepatic malignancies. It accounts for 80% of cases [1, 2]. Besides viral hepatitis, excessive consumption of alcohol, diabetes/obesity are important risk factors of HCC, especially in the western world [3]. Early HCCs, which are limited to the liver, can be treated with curative intend through either surgical resection (LR) or liver transplantation (LT) [4]. Liver resection leaves precancerous cirrhotic liver tissue in the patient and is also limited by the remaining functional liver volume. Both factors contribute to early recurrences and a relative high morbidity and mortality [5]. LT offers a complete resection of the precancerous tissue with an ideally immediately reconstituted liver function. However, livers for transplantation are scarce and LT associated risks are high since patients have to be treated with immunosuppression the rest of their lives [6].

When considering these differences, it is clear that accurate prediction of tumor specific survival after resection is needed. Until now mostly retrospective studies offered clinical prediction, which could not be confirmed in independent test cohorts [4, 7]. Besides novel statistical techniques, some groups of variables show great promise for preoperative prediction of disease-free survival after resection [8]. Increasingly, the antitumor immune response in the cancerous tissue (tumor infiltrating leukocytes; TILs) has been studied as a relevant predictor for survival after HCC resection and transplantation [9–11]. The measurement of TILs, however, is only possible after the resection of the tumor or would require a biopsy, which is invasive and could prove to be dangerous in cirrhotic patients [12]. Up-to-now some studies have measured circulating immune cells in HCC patients. These studies, however, investigated only small subsets of immune cells. Additionally, mostly either the immune activating or the immune suppressing axis of the antitumor response is elucidated [13]. This, however, does not give a complete, comparable and standardized view of the immunity in patients with HCCs. Furthermore, the measurement of the tumor immune response is influenced by the underlying liver disease since most HCCs develop in hepatitis patients [14]. In hepatitis infiltrating immune cells are in part complicit in the initiation and progression of HCC. A complete characterization of the tumor immunity against HCC in non-HBV/non-HCV patients, however, has not yet been attempted. With data science methods vast amounts of data can be analyzed and compared. Rather than comparing each and every single cell group or marker, dimensional reduction techniques such as principal component analyses (PCA) allow for explorative grouping of signatures. With this visualization of complex patterns in multi-marker models is possible [15].

For bacterial infections or leukemic diseases, the differential white blood cell count (DIF) is used to diagnose and monitor the disease. While the DIF has been shown to react to the presence of a malignant tumors, the resolution of it is not high enough to accurately measure the antitumor response against HCC, especially in non-HBV/non-HCV patients [16].

Therefore, in this study we measured a highly differential leukocyte count (hDIF) using multicolor flow cytometry (FCM) in non-HBV/non-HCV patients. The immune signatures were compared to healthy donors. Additionally, we measured TILs with the same technique and panels to show the differences in immune response in PB and tumor tissue. Furthermore, we demonstrated the functionability of the major immune effector cells infiltrating into HCC.

## Material and methods

### Patient and healthy donor selection

This study was planned to truly investigate the effects of hepatocellular carcinoma on the patients’ immune response without the immunological effects of apparent cirrhosis or hepatitis related disease. Therefore, in this experiment, 10 patients with primary hepatocellular carcinoma were enrolled in this study. None of these patients had hepatitis *B* virus or hepatitis *C* virus infection. All the patients underwent surgical treatment from at the Ludwig-Maximilians-University Munich (LMU) hospital. Ten healthy volunteers were used as healthy controls. With the collection of specimens, we obtained the informed consent of the volunteers. Institutional review board approval and registration was obtained (#EK 54–16, 53–16, DRKS00013567). Epidemiological data and clinical characteristics including gender, age and cirrhosis grade were collected in our database.

### Identification and staining of immune cells

Supplemental Table 1 shows all cell subsets and their cluster of differentiation (CD). The flow cytometric analyses were designed in a modular system comprising 4 different panels which examine *T* cells, *B* cells, monocytes, neutrophils, dendritic cells (DC), MDSC, NK and NKT cells and IFN-*γ*. As standard, each panel included unstained tubes which served as blank control, FMO control tubes and experimental tubes.

### Measurement procedure

For measurement of peripheral blood (PB) samples were collected from healthy donors and HCC patients before operation. All specimens were tested as soon as possible and never after longer than 24 h after harvesting. All experiments were conducted at room temperature. Whole blood was measured directly.

Tumor infiltrating leukocytes (TILs) were isolated out of fresh HCC tissues after surgery. 10 ml collagenase was added. After shaking and addition of 5 ml Trypsin EDTA (Lonza, Basel, Switzerland), the mixture was pressed through 100 µm filter. The collected mixture was centrifuged 500xg for 5 min. 10 ml ACK buffer (Fluka Inc., USA) and sequentially PBS Puffer was added and the mixture was pressed through 40 µm filter repeatedly. A portion of collected tumor infiltrating cells was measured directly after cell counting. The other cells were used to detect the secretion of IFN-*γ*. These cells were divided into two groups, stimulated cells and non-stimulated cells group. BD leucocyte activation kit (BLK) (Becton Dickinson Inc., USA) and Golgi stop (Becton Dickinson Inc., USA) were added and incubated. FACS DIVA™ Software (Becton Dickinson Inc., USA) was used to analyze the obtained data and gate the results.

### The in-vitro co-culture system

Tumor tissue specimens were cut into 2–4 mm pieces and treated with tumor dissociation kit (Miltenyi Biotec, Germany) for long-term storage. MACS was applied to separate CD45^+^ cells (TILs) and CD45-cells (primary HCC cells) following the protocol. The co-culture step was the same as HepG2-PBMC co-culture mentioned before. A ratio of 1:10 (Primary HCC cells:TILs) was finally adopted, and FACS analysis was applied at 0 h and 24 h. FVS 510 was added for viability test and CD107a for degranulation level measurement. Gating strategy of major immune effector cells is the same as described before. As a technical control for the co-cultivation of TILs and tumor tissue we employed in-vitro co-culture system of HCC cell line HepG2 and PBMC. HCC cell line HepG2 cells were seeded into a 6-well plate 6 h before co-culture with 3 mL of co-culture medium (DMEM: F12 with 10% FBS and 1% P/S) per well to allow them attaching to the bottom. Freshly isolated PBMCs were then added into the well with the ratio of 1:10 or 1:25 (HepG2:PBMC). They were incubated in the co-culture incubator (37 °C, 5% CO2) for 24 h. Same amount of HepG2 cells and PBMCs were cultured alone with the same conditions as two control groups. 24 h later, cells in the supernatant were collected as PBMC. Cells attached to the bottom including HepG2 cells and part of PBMCs can be detached by Trypsin/EDTA and distinguished by FACS. FACS analysis was applied at 0 h and 24 h.

### Statistical analysis

SPSS software package (Version 21.0, IBM Corp., USA) was used for statistical analysis. The Kolmogorov–Smirnov method was used to test whether the measurement variables were subjected to normal distribution; normally distributed variables and non-normal distributed variables were compared with unpaired *T*-test and Mann–Whitney *U* test. Paired *T*-test was used to compare results from peripheral blood and tumor tissue of the same patient and IFN-*γ* secretion. One-way ANOVA (One-way analysis of variance) was used for the viability and degranulation comparison in co-culture system. A *P*-value less than 0.05 was considered statistically significant.

For multidimensional analysis RStudio (Version 1.1.463, RStudio Inc.,USA) and following packages were used: ggplot2, FactoMineR and factoextra [15, 17].

Missing data was calculated using the missing Forest algorithm. The PCA Biplot was drawn showing the clustering of individuals and the contribution to this clustering from each variable.

## Results

### Patients and baseline data

For this study peripheral blood samples age and gender matched of 10 HD (6 male and 4 female) and 10 HCC (7 male and 3 female) patients were examined. HD were 61.2 ± 10 and HCC patients 65.95 ± 12.9 old. Patients included showed no signs of cirrhosis and therefore were scheduled for resection. However, 4 out of 10 (40%) suffered from non-alcoholic fatty liver disease (NAFLD). None of the patients received prior treatment or had any history of malignant disease. No statistical significance in age and gender distribution could be detected. As per the protocol three extensive panels were measured in the PB and TILs. The detailed results of all subsets of cells are listed in supplementary Tables 1 and 2. In the following the relevant cell groups and subsets which illustrate the state of the antitumor response are described.

### Highly Differential Leukocyte Count (hDIF) of Healthy Donors and Hepatocellular Carcinoma Patients is able to represent Activation and Suppression of the Antitumor Response

**Fig. 1:**
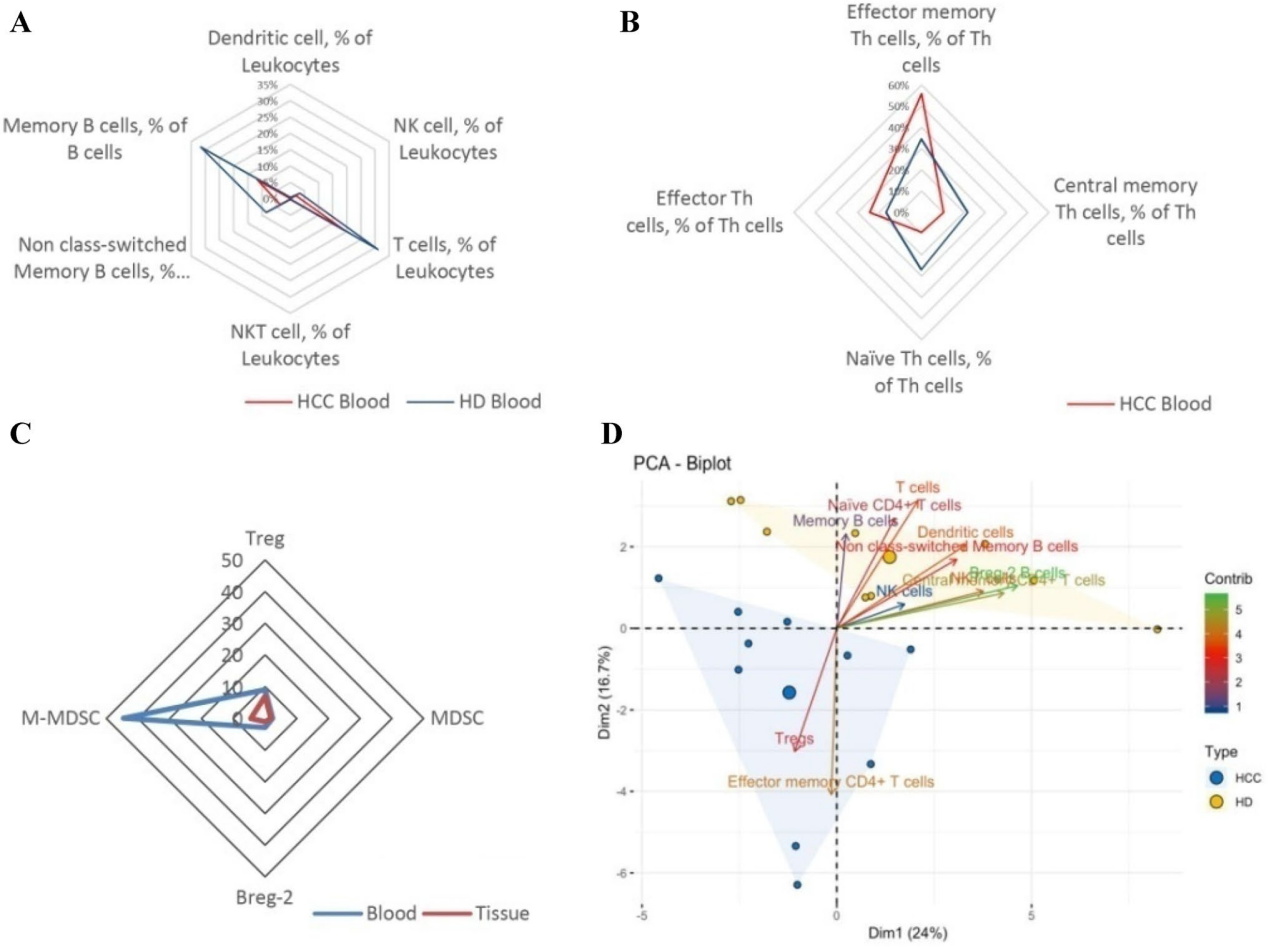
**a** Spider graph of immuno-activating cells; **b** Spider graph of corresponding Th subgroups; **c** Spider graph of immuno-suppressing cells; **d** Biplot of the PCA of all individuals

**Fig. 2:**
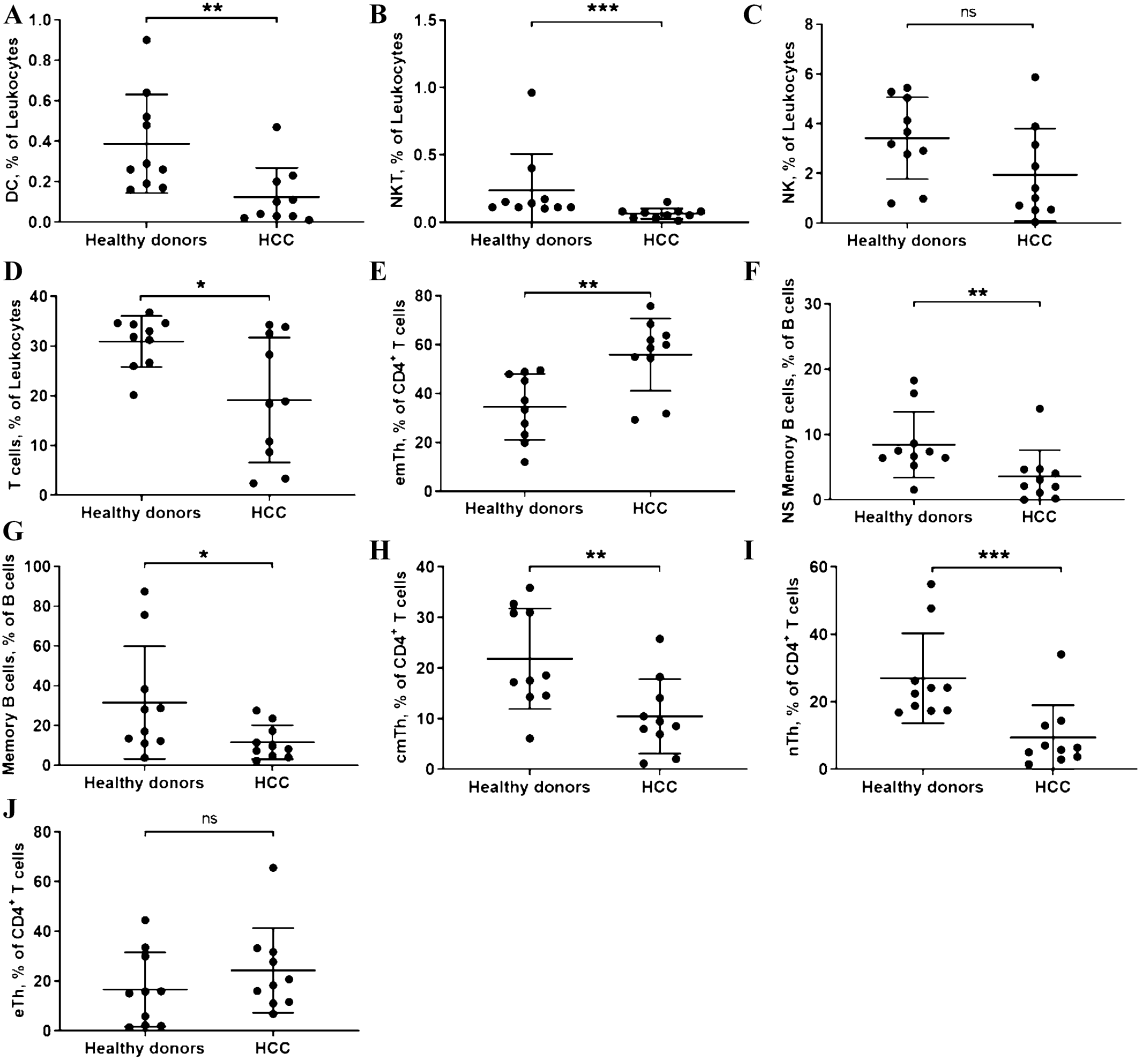
Comparison of DC, NKT, NK, *T* cells, subsets of Th, ns-memory *B* cells and Memory B cells in HD and HCC patients. (**a**: DC; **b**: NKT; **c**: NK; **d**: *T* cells; **e**: emTh cells; **f**: Ns-memory *B* cells; **g**: Memory *B* cells; **h**: cmTh cells; **i**: nTh cells; **j**: eTh cells. *: *P* < 0.05; **: *P* < 0.01; ***: *P* < 0.001; *ns* no significance.)

Cells that activate or suppress the antitumor response can themselves be expanded/stimulated and inhibited. First, we will describe the antitumor response activating cells and their changes due to the presence of the tumor. Figure 1a is a summary of the relevant immune activating measured cell subsets. Detailed analyses and representative flow-cytometric images are provided in Fig. 2 and supplementary Fig. 1. Most cells from the innate immune system have no positive antitumor effect. However, DCs and NK-cells have been reported to have relevant antitumor activity [18, 19]. As shown in Fig. 1a DCs in HD were 0.317 ± 0.076% and in HCC patients 0.124 ± 0.045% (*p* = 0.009). Also illustrated in the figure, NK-cells were less frequently measured in HCC patients than in HD (HCC patients: 1.943 ± 0.59% vs. HD: 3.42 ± 0.52%; *p* = 0.08).

T cells in general showed lower percentages in PB of HCC patients (HCC patients: 19.15 ± 3.97% vs. HD: 30.93 ± 1.62%; *p* = 0.0013; Fig. 1a). The ratio between CD4^+^ and CD8^+^ cells was not changed by the presence of the HCC (Supplementary Table 2). However, when looking at the subsets of T cells, a shift toward more effector memory *T* cells could be observed (HCC patients: 55.92 ± 4.67% vs. HD: 34.54 ± 4.26%). This difference was significant at *p* = 0.003. At the same time the corresponding subgroups naïve *T* and central memory *T* cells showed lower percentages in HCC patients (Supplementary Table 2). The differential analysis is depicted in Fig. 1b, which illustrates the shift in *T* cell subsets. A special group is NKT cells. These cells were also less frequent in HCC patients (HCC patients: 0.06 ± 0.01% vs. HD: 0.24 ± 0.27%; *p* < 0.001; Fig. 1a).

Compared to HD *B*-cell distribution changed as well in the presence of HCC. Nonclass-switched memory *B* cells (HCC patients: 3.59 ± 1.27% vs. HD: 8.44 ± 1.6%; *p* = 0.005) and memory *B* cells (HCC patients: 11.6 ± 2.69% vs. HD: 31.57 ± 8.96%; *p* = 0.047) were less frequent in HCC patients. This is also depicted in Fig. 1a.

**Fig. 3:**
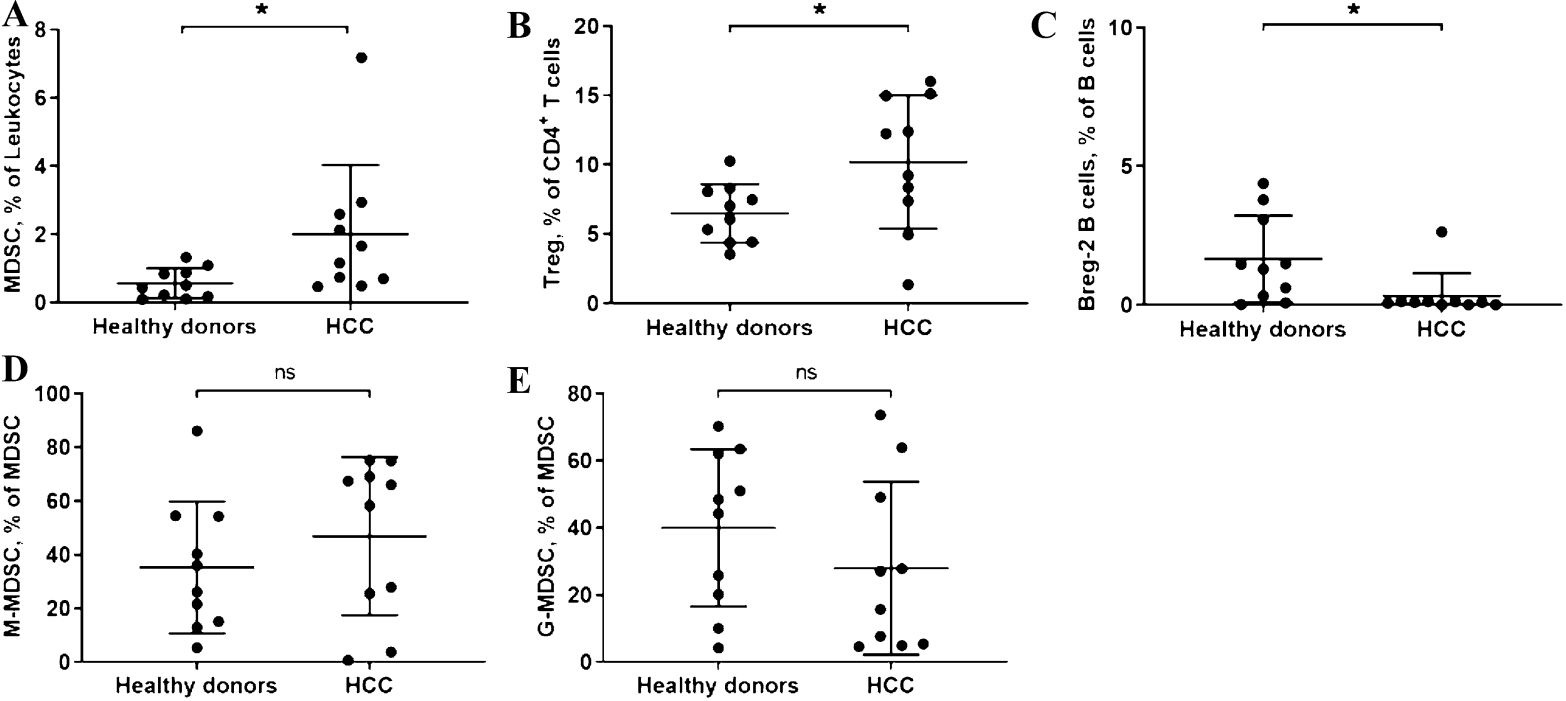
Comparison of MDSC, Treg and Breg-2 in HD and HCC patients. (**A**: MDSC; **B**: Treg; **C**: Breg-2; D: M-MDSC; E: G-MDSC. *: *P* < 0.05; *ns* no significance.)

Second, the antitumor response suppressing cells also showed a stimulation and inhibition by the HCC. The reaction of the mostly immunosuppressive cells is summarized in Fig. 1c. The detailed analyses and representative flow-cytometric images are shown in Fig. 3 and supplementary Fig. 2. MDSCs (HCC patients: 2.005 ± 0.64% vs. HD: 0.564 ± 0.139%; *p* = 0.041) showed themselves to have a higher percentage in HCC patients. In contrast to that M-MDSCs and G-MDSCs remained unchanged (Fig. 1c). Regarding T cells, the known immunosuppressive cells Tregs were significantly accumulated in HCC-Patients (HCC patients: 10.2 ± 1.52% vs. HD: 6.49 ± 0.67%; *p* = 0.038) (Fig. 1c). No differences in the Treg subgroups were detected (Supplementary Table 2). Even though the role of Breg cells (defined as: CD24hiCD38^+^CD1d^+^CD5^+^) in the antitumor defense has not yet been fully uncovered, we could measure a significant difference in circulating Bregs in HCC patients compared to HD (HCC patients: 0.32 ± 0.26% vs. HD: 1.65 ± 0.5%; *p* = 0.014) (Fig. 1c, Fig. 3 and supplementary Fig. 2).

As mentioned above, dimensional reduction allows for the grouping of immune signatures. For this we drew a Biplot of the PCA of all individuals as well as the contribution of the significantly different variables. With this it can be seen that immune activating cells contribute to the differences in immune cells distribution between HCC patients and HD (Fig. 1d).

### Comparison of circulating immune-cells and the tumor infiltrating leukocytes illustrates an antitumor activating gradient

**Fig. 4:**
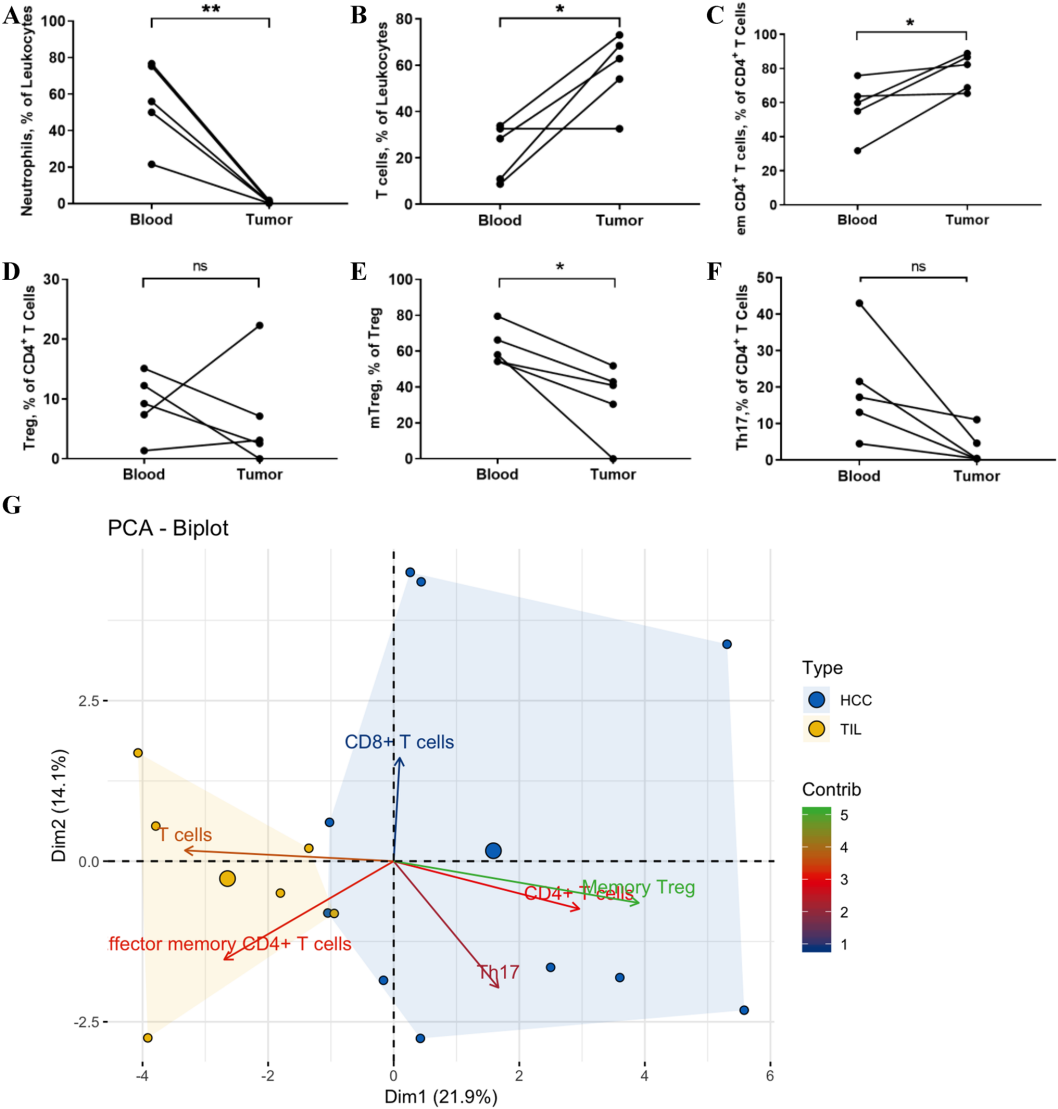
Comparison of immune cells in freshly harvested HCC blood and tissue. (**a**: Neutrophils; **b**: *T* cells; **c**: em CD4 + *T* cells; **d**: Tregs; E: mTregs; **f**: Th17 cells; **g**: Biplot of the PCA of all individuals as well as the contribution of the single variables. *: *P* < 0.05; **: *P* < 0.01; *ns* no significance.)

Relevant results from the comparison of circulating immune-cells and the tumor infiltrating leukocytes are depicted in Fig. 4. Neutrophil granulocytes, one of the most abundant cell groups, that have been described as inhibiting the antitumor response when infiltrating in the tumor, showed less percentages than in the PB (PB: 55.92 ± 10.07% vs. TILs: 0.85 ± 0.25%; *p* = 0.005; Fig. 4a). Consequently, in comparison *T* cells are much more frequent in the tumor (PB: 42.61 ± 6.70% vs. TILs: 58.17 ± 7.32%; *p* = 0.021; Fig. 4b). There was no significant difference in the CD8^+^ *T* cells and their subpopulations between HCC tissues and peripheral blood. Compared with peripheral blood, the ratio of CD4/CD8 decreased significantly in the tissue (*p* = 0.024). Regarding the subgroups, similar to the PB, showed a shift toward effector memory *T* cells (PB: 57.28 ± 7.24% vs. TILs: 78.43 ± 4.77%; *p* = 0.042; Fig. 4c).

Immunoregulatory and immunosuppressive cells were also present in the HCC tissue. The percentage of tumor infiltration Tregs was not statistically different from that in peripheral blood. This is shown in Fig. 4d. However, within the Treg subgroup memory Treg cells in tumor tissues were significantly lower accumulated than in peripheral blood (PB: 62.53 ± 4.79% vs. TILs: 33.25 ± 8.99%; *p* = 0.018; Fig. 4e). There were no statistically significant differences in the other three subgroups of Treg (Supplementary Table 3). The proportion of Th17 showed a decreasing trend in HCC tissue compared to PB (*p* = 0.057) (Fig. 4f). No difference in B cell distribution could be detected between the peripheral blood and HCC tissue.

These results are shown in detail in Fig. 4a–f and supplementary Fig. 3. As with the comparison of HCC patients with HD a Biplot of the PCA of all individuals with the corresponding tumor tissue as well as the contribution of the significantly different variables was drawn. In this it can be seen that PB and TILs form distinctive signature cluster. The immune activating cells contribute to the differences in clustering between PB and TILs (Fig. 4g).

### Tumor infiltrating leukocytes showed functional capability by secreting IFN-γ but were not able to kill HCC cells

**Fig. 5:**
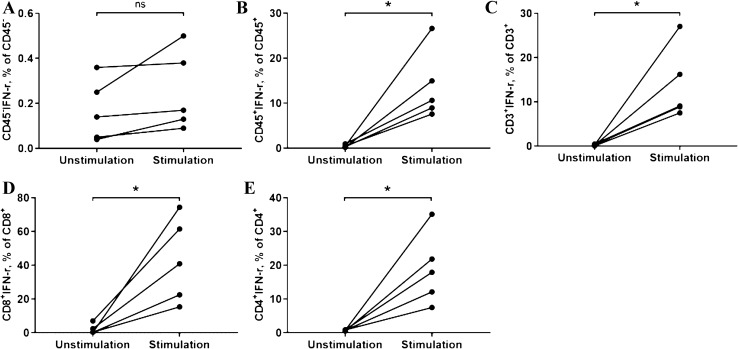
Comparison of IFN-*γ* in unstimulated group and stimulated group. (**a**: IFN-γ of CD45- cell populations; **b**: IFN-*γ* of CD45 + cell populations; **c**: IFN-γ of CD3 + cells; **d**: IFN-*γ* of CD8 + cells; **e**: IFN-*γ* of CD4 + cells; *: *P* < 0.05; *ns* no significance.)

IFN-γ secretion was measured in CD45^+^, CD3^+^, CD4^+^ and CD8^+^ using flow cytometry. After stimulation all cell groups showed a significant increase in IFN-*γ* secretion. In CD45^+^ leukocytes, the percentage of IFN-*γ* in the stimulation group was (13.75 ± 6.88%) and in the non-stimulation group (0.49 ± 0.26%) (*p* = 0.019) (Fig. 5b). The secretion of IFN-γ from CD3^+^ *T* cells in stimulation group was 13.76 ± 7.31%, and the non-stimulation group 0.26 ± 0.14% (p = 0.021) (Fig. 5c). Similarly, the secretion of IFN-*γ* from CD4^+^ T cells was significantly higher than that in the non-stimulation group (Fig. 5e). CD8^+^ cells, defined by the secretion of IFN-γ, were as well stimulated and showed a significant increase (unstimulated: 2.03 ± 2.94% vs. stimulated: 42.93 ± 25.07%; *p* = 0.019) (Fig. 5d). Typical FCM pictures are shown in supplementary Fig. 4.

Simultaneously cytotoxicity was measured using a co-culture. Although the technical control was positive with a reduced viability and significantly increased degranulation. In the *co-culture* system (primary HCC cells and TILs), no difference of cytotoxicity was observed between co-cultured group and the control groups. The viability of primary HCC cells (CD45^−^) showed no difference (Supplementary Fig. 5, 6a). No difference regarding degranulation level of CD8^+^ T lymphocytes and NK cells was found either (Supplementary Fig. 6b, c and 7).

## Discussion

The immune response to a malignant tumor has been widely investigated. Since the introduction of immunoscoring for colorectal carcinomas, clinicians and researches have been inspired to develop similar scoring systems for many solid tumors [9, 20–22].

While being predictive for a variety of solid malignancies, these scoring systems can only be measured after the operation, i.e. after the treatment decision has been made. Especially since HCC can be treated with two distinctively different surgeries, LR and LT, a predictive immunoscoring before the treatment decision has been made, would be ideal. [12, 23, 24].

With this we introduce the concept of a hDIF for the measurement of circulating immune cells in case of HCC and compare the results to healthy donors (HD) and tumor infiltrating leukocytes (TILs). We intentionally concentrated on the small group of non-HBV/non-HCV HCC with no apparent cirrhosis to better approximate the true immune response against HCC and not the underlying disease. We analyzed 39 subsets of immune cells including *T* cells, *B* cells, monocytes, neutrophils, dendritic cells, MDSC, NK and NKT cells. Even though this large number of cell groups was measured, some important and emerging cell subsets had to be excluded. Besides the already included *T* helper cells (Th1, Th2, Th17, *T* regs and subsets) novel subsets such as Th9 and Th22 hold the potential to influence HCC tumor immunity [25]. By concentrating on the aforementioned subsets, we felt that we had the broadest spectrum of cells to cover the tumor immunity of HCC. It is the first study that analyzes immune activating and suppressing cells alike to put them into perspective without influence of hepatitis.

Analyses of the innate immune system are scarce. Information on circulating DC cells is not available in the literature. In this study we found that those were significantly more frequent as compared with HD. Information on NK-cells in published studies is conflicting. Atallah et al. and other groups have reported that NK cells are more frequent in HCC patients [26, 27]. Confirming our results Cai et al., however, showed a decrease of circulating NK-cells [28]. The measurement of NK cells even showed an influence on survival and a functional impairment [18]. The work by Attalah et al. was done in patients suffering from schistosomiasis and hepatitis patients, whereas (similar to this work) Cai et al. investigated patients without a history of hepatitis. NK cells play a major role in patients’ response to viral hepatitis (Hepatitis *B* and *C* alike) especially in the early phases. NK-cells which are CD56 + bright have been reported to have a direct cytotoxic effect on infected hepatocytes. In chronic hepatitis NK-cell prevalence and function is reduced [29, 30]. Regarding immune suppressing cells the most analyzed are MDSCs. This cell group correlated with CTP score and disease advancement in the literature [31]. Despite the patients not having measurable cirrhosis in our work MDSC frequencies in the PB were significantly higher in HCC patients, suggesting a recruitment of these immunosuppressive cell subsets in case of HCC alone.

Regarding the adaptive immune cells much more results from the literature are available. Although plentiful, many publications are conflicting. In overall regarding *T* cell measurement, we found significantly less percentages as compared with HD. This is confirmed by Attallah et al. [26]. In hepatitis *B* positive patients, it has been reported that CD4^+^ cells were less frequent but not CD8^+^ cells. This too was measured in patients with a history of hepatitis. This virus-associated immunosuppression was not present in our cohort [32, 33]. When differentiating into the subgroups of CD4^+^ positive cells, we could detect significant shift toward emTh cells. This effect is different when compared to hepatitis patients, as could be shown by a very recent study [27]. Our study was the first study to measure a significant decrease in NKT cell frequency in the PB, whereas two studies in the literature suggested no change and one a significant increase. These studies, however, analyzed patients with hepatitis *B* or *C* which when chronic exerts an immunosuppressive environment as mentioned above [27, 30, 32, 34].

Circulating *B* cells in general have not been studied well but were put into context using our hDIF. In here we could show a decrease in non-class-switched memory *B* cells and memory *B* cells. These results are confirmed by one study that additionally suggested that the amount of memory *B* cells decreases with tumor progression [35]. Due to the small sample size, we cannot confirm this interesting finding. *B* regs are a newly discovered subset of *B* cells that similarly to *T* regs show an immune regulatory effect. In our cohort, we detected a significant reduction in circulating *B* regs (defined as: CD24^hi^CD38^+^CD1d^+^CD5^+^). Chen et al. also reported on a decrease of *B* regs [36]. In their study, however, this distinctive and not very frequent cell group was defined as CD19^+^ IL-10^+^. This study also reported on a positive correlation with HBV DNA copies as well as hepatitis E Antigen [36]. However, in combination with our results this suggests that *B* regs play a role in HCC immunity independent from the underlying disease. Further studies are needed to understand the function of B regs in this context and to identify potential treatment targets.

When analyzing the changes in distribution of PB immune cells certain subsets seem to be influenced by the presence of HCC. With the broad spectrum of cells that were measured we now can identify the most relevant immune cells for future studies. The relevant immune cells can be measured with significantly less fluorochromes thereby saving blood to be drawn from patients, increasing efficiency, allowing for more flexibility toward new cell subsets and lastly saving money. We believe future studies when investigating the immune activating axis should concentrate on: *T* cells (both CD4^+^ and CD8^+^), effector memory *T* cells, central memory *T* cells, naïve *T* cells, NK-cells, NKT cells, dendritic cells and non-class-switched Memory *B* cells. When investigating the immunosuppressive axis in the PB future studies should consider: *T* regulatory cells, MDSC, Th17 cells and finally the newly described B regs (CD24^hi^CD38^+^CD1d^+^CD5^+^).

Additionally, to the hDIF from PB we compared the TILs to the corresponding PB samples to describe the delineation of these two reservoirs. This is especially important to see whether the hDIF of the PB is suitable to illustrate the immune response in the tumor. To our knowledge only two recent publications have examined the delineation between the PB and TILs in HCC. The work by Chew and colleagues only included 19% non-HBV/non-HCV patients, the researchers found an immunosuppressive gradient and significant exhaustion of the tumor infiltrating immune cells [14]. This was also confirmed by another study most recently published with only hepatitis patients [27]. Our study shows a relevant immune activation in the tumor by a significant higher infiltration rate of immune activating cells. First of all, the immune suppressive CD66b^+^ neutrophils are less frequent in the tumor [37]. By that alone the relative ratio of *T* cells was significantly higher in the tumor. More so, the shift toward effector memory *T* cells is also present in the tumor such as in the PB. The proportion of other cell groups showed almost no differences between the PB and TILs. Those cells which infiltrated the tumor itself could still be activated. CD45^+^, CD3^+^, CD4^+^, CD8^+^ cells secreted IFN-*γ* as a sign of their responsiveness. These differences with the remarkable work of Chew et al. and Chaoul might again be related to the lack of an underlying viral disease and no measurable cirrhosis. Other work has already indicated that HBV-related HCCs induce a more immunosuppressive environment than non-HBV/non-HCV HCCs [38]. This seems to also hold true for the so-called invasive margin (or nontumor microenvironment (NTME)) of the HCC. In the previously discussed cohort of mostly hepatitis patient both invasive margin and tumor showed relevant (but not to the same degree) immune exhaustion [14]. In a previous study regarding TILs, we introduced the perivascular region as a potentially more relevant border to the patients’ immune system [9]. In fact, due to the high degree of angiogenesis of HCC, the surface area of the vessels is the largest border from tumor to the immune system [39].

The fact that TILs accumulating have similar percentages as circulating immune cells in PB shows that a highly differentiated measurement of the PB could represent the immune reaction that is present in the tumor itself.

This study shows limitations regarding the number of patients recruited for this analysis. In order to measure the true effects against HCC we opted to include patients without apparent cirrhosis. Therefore, the results were also compared to only age and gender matched individuals. Even though the overall number of participants is low, we could show robust results that are also supported by the literature. Furthermore, in Europe HBV and HCV HCC are still frequent and were excluded for this study to report on a homogenous group of patients without the immune modulatory effect of the chronic infection and subsequent cirrhosis. It should be mentioned that 40% of our patients had NAFLD, which might influence the results slightly since NAFLD promotes STAT3 in tumor cells mediated through effector and helper *T* cells which might promote cancer initiation. However, we could show the anti-tumor effect of both cell groups. The detail of this interplay needs to be investigated by studies concentrating on the etiology and development of non-HBV/non-HCV HCC. Lastly, because of the highly differentiated measurements in this study it was not always possible to isolate enough cells to measure. With the results from this study however, we will be able to concentrate on the relevant cell groups and add additional cell groups that might also be interesting to investigate.

With an automated and global statistical analysis using multidimensional reduction and contribution, it was possible to analyze the entirety of a vast amount of immune cells. This kind of analysis has not been attempted in non-HBV/non-HCV HCC patients before [14, 38].

In conclusion, we can show that the measurement of a highly differentiated blood cell count (hDIF) shows distinct differences in immune reactions against non-HBV/non-HCVHCC as compared to HD. In this analysis, non-HBV/non-HCV HCC triggered an immune response with an immunosuppressive gradient toward peripheral blood.

## Supplementary Information

Below is the link to the electronic supplementary material.

Supplementary file1 (DOCX 7502 KB)

Supplementary file2 (DOCX 35 KB)

## Declarations

### Data availability

Data will be made available upon reasonable request to the corresponding author.

## References

[1] European Association For The Study Of The L, European Organisation For R, Treatment Of C (2012) EASL-EORTC clinical practice guidelines: management of hepatocellular carcinoma. J Hepatol 56(4):908–943 10.1016/j.jhep.2011.12.001 22424438

[2] Torre LA, Bray F, Siegel RL, Ferlay J, Lortet-Tieulent J, Jemal A (2015). Global cancer statistics, 2012. CA Cancer J Clin.

[3] Morgan TR, Mandayam S, Jamal MM (2004). Alcohol and hepatocellular carcinoma. Gastroenterology.

[4] Schoenberg M, Bucher J, Vater A, Bazhin A, Hao J, Guba M, Angele M (2017). Resection or transplant in early hepatocellular carcinoma—a systematic review and meta-analysis. Dtsch Ärztebl Int.

[5] Malek NP, Schmidt S, Huber P, Manns MP, Greten TF (2014). The diagnosis and treatment of hepatocellular carcinoma. Dtsch Arztebl Int.

[6] Adam R, Azoulay D, Castaing D, Eshkenazy R, Pascal G, Hashizume K, Samuel D (2003). Liver resection as a bridge to transplantation for hepatocellular carcinoma on cirrhosis: a reasonable strategy?. Ann Surg.

[7] Schoenberg MB, Anger HJW, Hao J, Vater A, Bucher JN, Thomas MN, Lauseker M (2018). Development of novel biological resection criteria for safe and oncologically satisfying resection of hepatocellular carcinoma. Surg Oncol.

[8] Schoenberg MB, Bucher JN, Koch D, Borner N, Hesse S, De Toni EN, Seidensticker M (2020). A novel machine learning algorithm to predict disease free survival after resection of hepatocellular carcinoma. Ann Transl Med.

[9] Schoenberg MB, Hao J, Bucher JN, Miksch RC, Anger HJW, Mayer B, Mayerle J (2018). Perivascular tumor-infiltrating leukocyte scoring for prognosis of resected hepatocellular carcinoma patients. Cancers.

[10] Brunner SM, Rubner C, Kesselring R, Martin M, Griesshammer E, Ruemmele P, Stempfl T (2015). Tumor-infiltrating, interleukin-33-producing effector-memory CD8(+) T cells in resected hepatocellular carcinoma prolong patient survival. Hepatology.

[11] Mei Z, Liu Y, Liu C, Cui A, Liang Z, Wang G, Peng H (2014). Tumour-infiltrating inflammation and prognosis in colorectal cancer: systematic review and meta-analysis. Br J Cancer.

[12] Sapisochin G, Goldaracena N, Laurence JM, Dib M, Barbas A, Ghanekar A, Cleary SP (2016). The extended Toronto criteria for liver transplantation in patients with hepatocellular carcinoma: a prospective validation study. Hepatology.

[13] Ormandy LA, Hillemann T, Wedemeyer H, Manns MP, Greten TF, Korangy F (2005). Increased populations of regulatory T cells in peripheral blood of patients with hepatocellular carcinoma. Cancer Res.

[14] Chew V, Lai L, Pan L, Lim CJ, Li J, Ong R, Chua C (2017). Delineation of an immunosuppressive gradient in hepatocellular carcinoma using high-dimensional proteomic and transcriptomic analyses. Proc Natl Acad Sci U S A.

[15] Kassambara A, Mundt F (2020). http://www.sthda.com/english/rpkgs/factoextra

[16] Ni XC, Yi Y, Fu YP, He HW, Cai XY, Wang JX, Zhou J (2015). Prognostic value of the modified glasgow prognostic score in patients undergoing radical surgery for hepatocellular carcinoma. Medicine.

[17] Le Sebastien JJ, Husson F (2008). {FactoMineR}: a package for multivariate analysis. J Stat Softw.

[18] Cariani E, Pilli M, Barili V, Porro E, Biasini E, Olivani A, Dalla Valle R (2016). Natural killer cells phenotypic characterization as an outcome predictor of HCV-linked HCC after curative treatments. Oncoimmunology.

[19] Cai XY, Gao Q, Qiu SJ, Ye SL, Wu ZQ, Fan J, Tang ZY (2006). Dendritic cell infiltration and prognosis of human hepatocellular carcinoma. J Cancer Res Clin Oncol.

[20] Miksch RC, Hao J, Schoenberg MB, Dotzer K, Schluter F, Weniger M, Yin S (2017). Development of a reliable and accurate algorithm to quantify the tumor immune stroma (QTiS) across tumor types. Oncotarget.

[21] Galon J, Costes A, Sanchez-Cabo F, Kirilovsky A, Mlecnik B, Lagorce-Pages C, Tosolini M (2006). Type, density, and location of immune cells within human colorectal tumors predict clinical outcome. Science.

[22] Atanasov G, Dietel C, Feldbrugge L, Benzing C, Krenzien F, Brandl A, Mann E (2017). Tumor necrosis and infiltrating macrophages predict survival after curative resection for cholangiocarcinoma. Oncoimmunology.

[23] Otto G, Herber S, Heise M, Lohse AW, Monch C, Bittinger F, Hoppe-Lotichius M (2006). Response to transarterial chemoembolization as a biological selection criterion for liver transplantation in hepatocellular carcinoma. Liver Transpl.

[24] Guba M, Angele M, Rentsch M, Jauch KW, Zachoval R, Kolligs F, Gerbes A (2013). Therapy of hepatocellular carcinoma before liver transplantation. Chirurg.

[25] Golubovskaya V, Wu L (2016). Different subsets of T cells, memory, effector functions, and CAR-T immunotherapy. Cancers.

[26] Attallah AM, Tabll AA, El-Sadany M, Ibrahim TA, El-Dosoky I (2003). Dysregulation of blood lymphocyte subsets and natural killer cells in schistosomal liver cirrhosis and hepatocellular carcinoma. Clin Exp Med.

[27] Chaoul N, Mancarella S, Lupo L, Giannelli G, Dituri F (2020). Impaired anti-tumor T cell response in hepatocellular carcinoma. Cancers.

[28] Cai L, Zhang Z, Zhou L, Wang H, Fu J, Zhang S, Shi M (2008). Functional impairment in circulating and intrahepatic NK cells and relative mechanism in hepatocellular carcinoma patients. Clin Immunol.

[29] Shabani Z, Bagheri M, Zare-Bidaki M, Hassanshahi G, Arababadi MK, Mohammadi Nejad M, Kennedy D (2014). NK cells in hepatitis B virus infection: a potent target for immunotherapy. Arch Virol.

[30] Zeromski J, Mozer-Lisewska I, Kaczmarek M, Kowala-Piaskowska A, Sikora J (2011). NK cells prevalence, subsets and function in viral hepatitis C. Arch Immunol Ther Exp.

[31] Wang D, An G, Xie S, Yao Y, Feng G (2016). The clinical and prognostic significance of CD14(+)HLA-DR(-/low) myeloid-derived suppressor cells in hepatocellular carcinoma patients receiving radiotherapy. Tumour Biol.

[32] Lin JC, Shih YL, Chien PJ, Liu CL, Lee JJ, Liu TP, Ko WC (2010). Increased percentage of B cells in patients with more advanced hepatocellular carcinoma. Hum Immunol.

[33] Liu HZ, Deng W, Li JL, Tang YM, Zhang LT, Cui Y, Liang XQ (2016). Peripheral blood lymphocyte subset levels differ in patients with hepatocellular carcinoma. Oncotarget.

[34] Li XF, Dai D, Song XY, Liu JJ, Zhu L, Zhu X, Ma W (2017). A different representation of natural T cells and natural killer cells between tumor-infiltrating and periphery lymphocytes in human hepatocellular carcinoma. Oncol Lett.

[35] Wang XD, Wang L, Ji FJ, Zhu JM, Ayana DA, Fang XD (2012). Decreased CD27 on B lymphocytes in patients with primary hepatocellular carcinoma. J Int Med Res.

[36] Chen T, Song D, Min Z, Wang X, Gu Y, Wei B, Yao J (2012). Perioperative dynamic alterations in peripheral regulatory T and B cells in patients with hepatocellular carcinoma. J Transl Med.

[37] Li YW, Qiu SJ, Fan J, Zhou J, Gao Q, Xiao YS, Xu YF (2011). Intratumoral neutrophils: a poor prognostic factor for hepatocellular carcinoma following resection. J Hepatol.

[38] Lim CJ, Lee YH, Pan L, Lai L, Chua C, Wasser M, Lim TKH (2018). Multidimensional analyses reveal distinct immune microenvironment in hepatitis B virus-related hepatocellular carcinoma. Gut.

[39] Muto J, Shirabe K, Sugimachi K, Maehara Y (2015). Review of angiogenesis in hepatocellular carcinoma. Hepatol Res.

