# Supplementary material for: Highly differential count of circulating and tumor infiltrating immune cells in patients with non-HCV/non-HBV hepatocellular carcinoma
Source: Cancer Immunol Immunother. 2021 Sep 28;71(5):1103–13. doi: 10.1007/s00262-021-03061-9 (PMC9015997; doi:10.1007/s00262-021-03061-9)
Supplement: Supplementary file 1 — Supplementary file1 (DOCX 7502 KB) [file 262_2021_3061_MOESM1_ESM.docx]

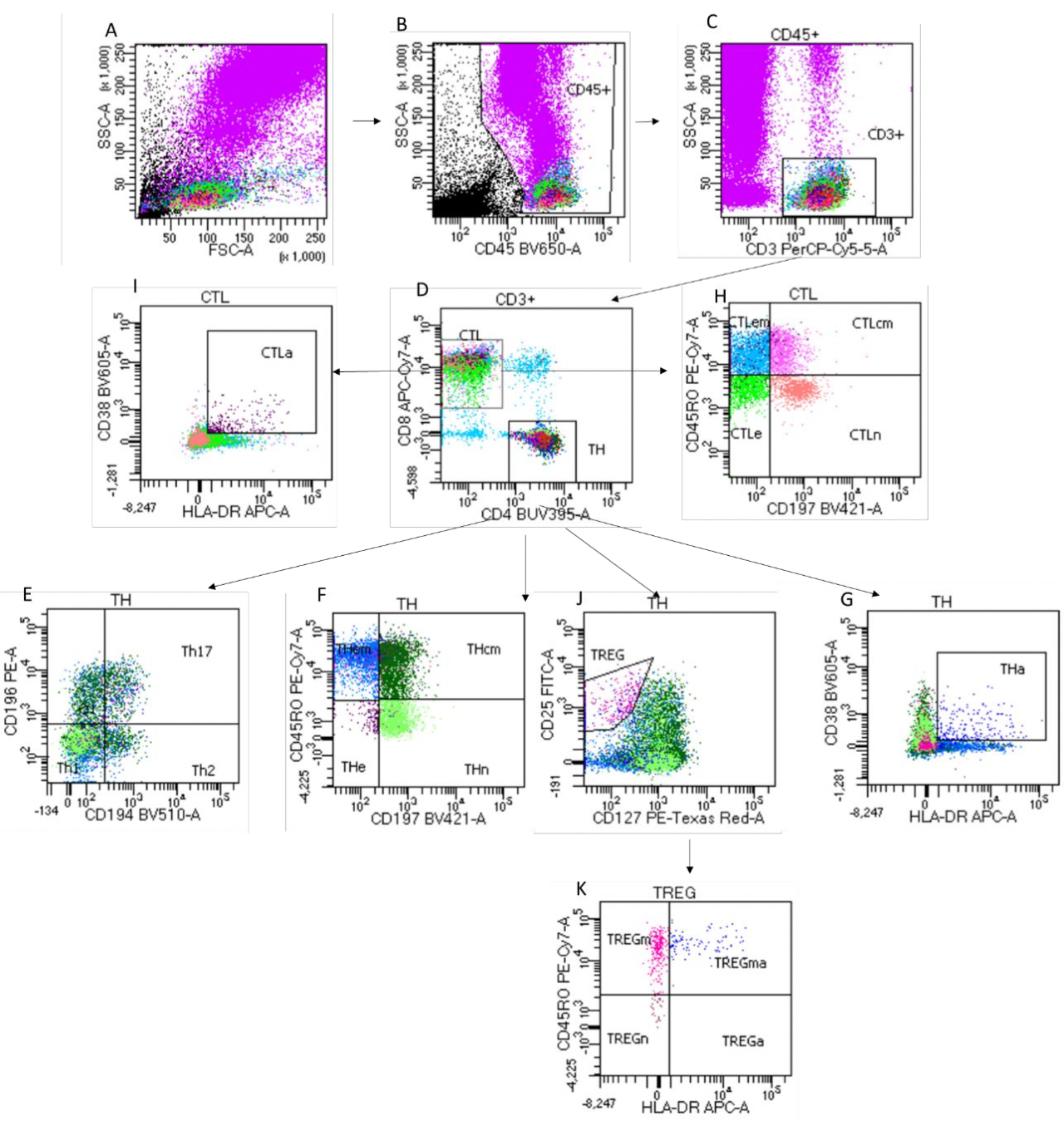


**Supplementary Figure 1:** Gating strategy for the T cell subsets. (A) All cells from PB except red blood cells; (B) Leukocytes; (C) CD3+ T cells; (D) According to differential CD4 and CD8 expression, Th and CTL were identified; (E) By CD194 and CD196 expressions, the Th were distinguished into Th1, Th2, and Th17; (F) According to differential CD197 and CD45RO expression, the Th were further distinguished into eTh, nTh, emTh, cmTh subsets; (G) CD38+/HLA-DR+ aTh; (H) According to differential CD197 and CD45RO expression, the CTL were further distinguished into eCTL, nCTL, emCTL, cmCTL; (I) CD38+/HLA-DR+ aCTL; (J) Tregs were identified by CD25+/CD127- expression; (K) By the CD45RO and HLA-DR expression, Tregs can be defined as naïve, memory, memory activated and activated Tregs.


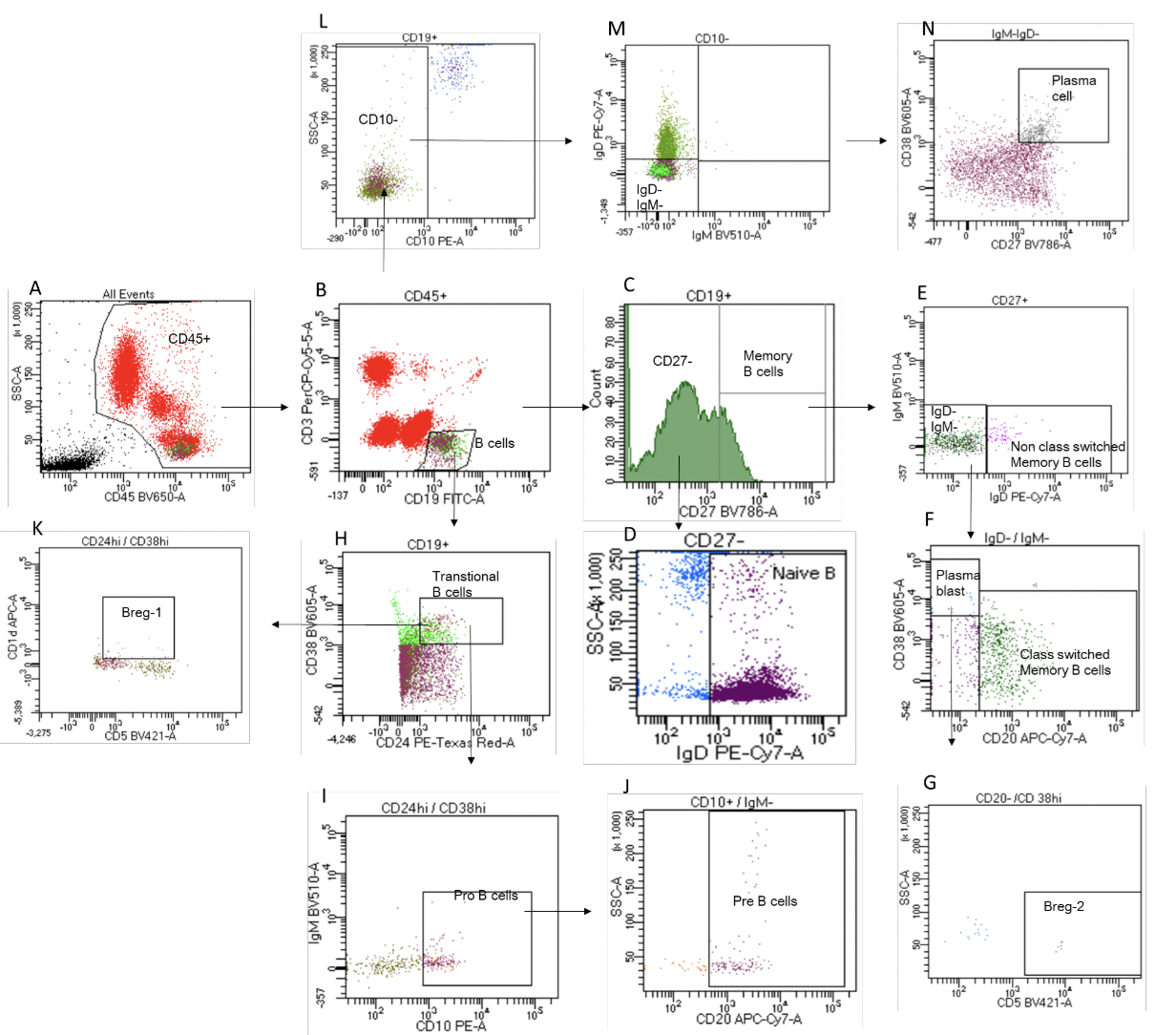


**Supplementary Figure 2:** Gating strategy for B cell subsets. (A) Leukocytes; (B) CD3- CD19+ B cells; (C) B cells were differentiated into CD27- populations and CD27+ memory populations; (D) Naïve B cells; (E) IgD- IgM- populations and IgD+ IgM- non class switched memory B cells; (F) Plasmablast and class switched memory B cells; (G) Bregs-2; (H) Transitional B cells; (I) Pro B cells from transitional B cells; (J) Pre B cells from pro B cells; (K) Regulatory B cells-1; (L) CD10- populations from B cells; (M) IgD- IgM- populations from CD10- populations of B cells; (N) Plasma cells.


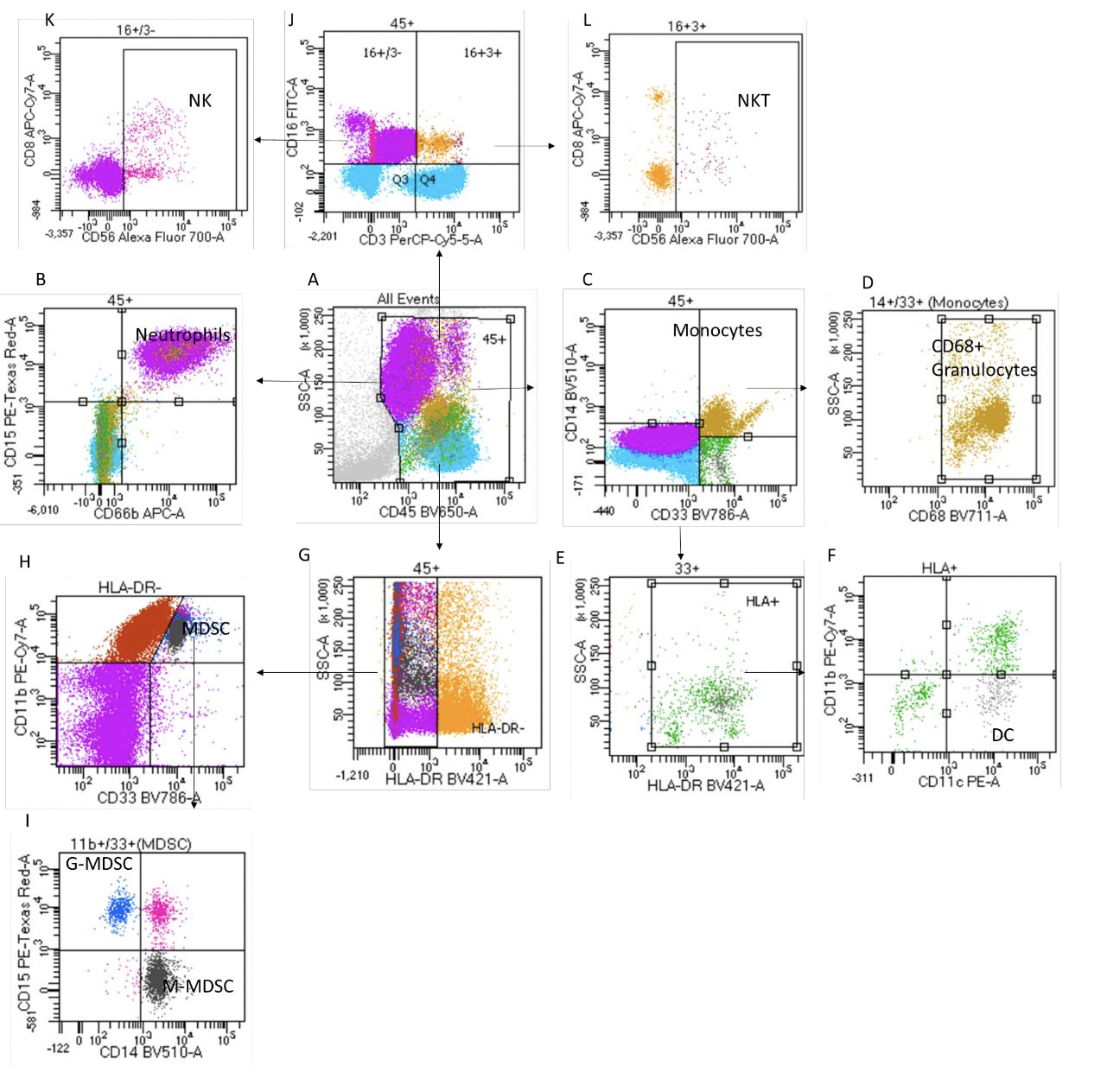


**Supplementary Figure 3:** Gating strategy for neutrophils, monocytes, macrophages and DC, MDSC. (A) Leukocytes; (B) Neutrophils; (C) Monocytes; (D) Macrophages; (E) HLA-DR+ subsets; (F) DC; (G) HLA-DR- subsets; (H) MDSC; (I) G-MDSC and M-MDSC; (J) CD3- CD16+ and CD3+ CD16+ subsets; (K) NK cells gated from CD3- CD16+ subsets; (L) NKT cells gated from CD3+ CD16+ subsets.


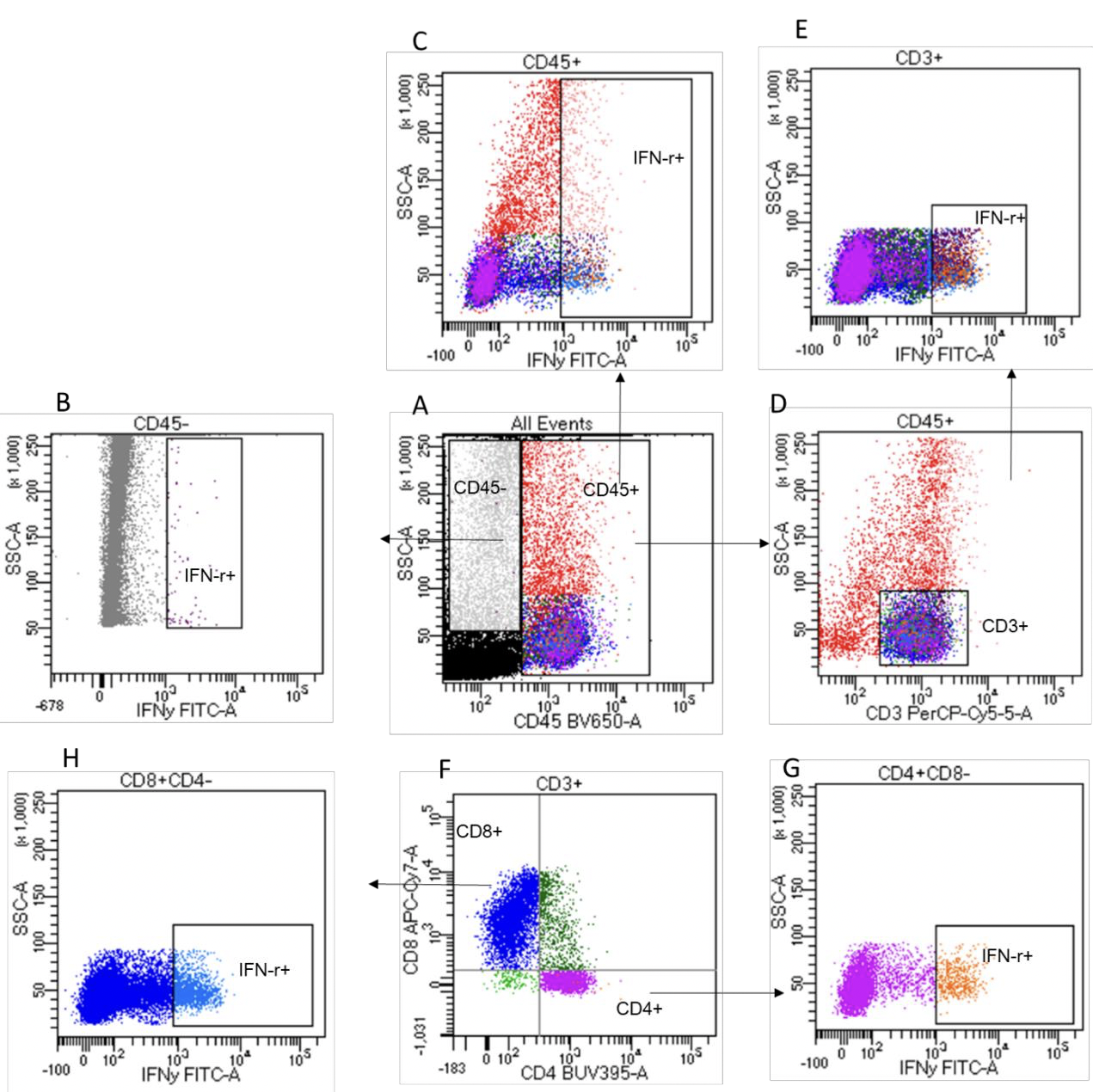


**Supplementary Figure 4:** Gating strategy for measuring IFN-γ in HCC tumor tissue. (A) Leukocytes and CD45- populations; (B) IFN-γ of CD45- populations; (C) IFN-γ of CD45+ leukocytes; (D) CD3+ T cells; (E) IFN-γ of CD3+ T cells; (F) CD4+ and CD8+ populations from T cells; (G) IFN-γ of CD4+ subsets; (H) IFN-γ of CD8+ subsets.


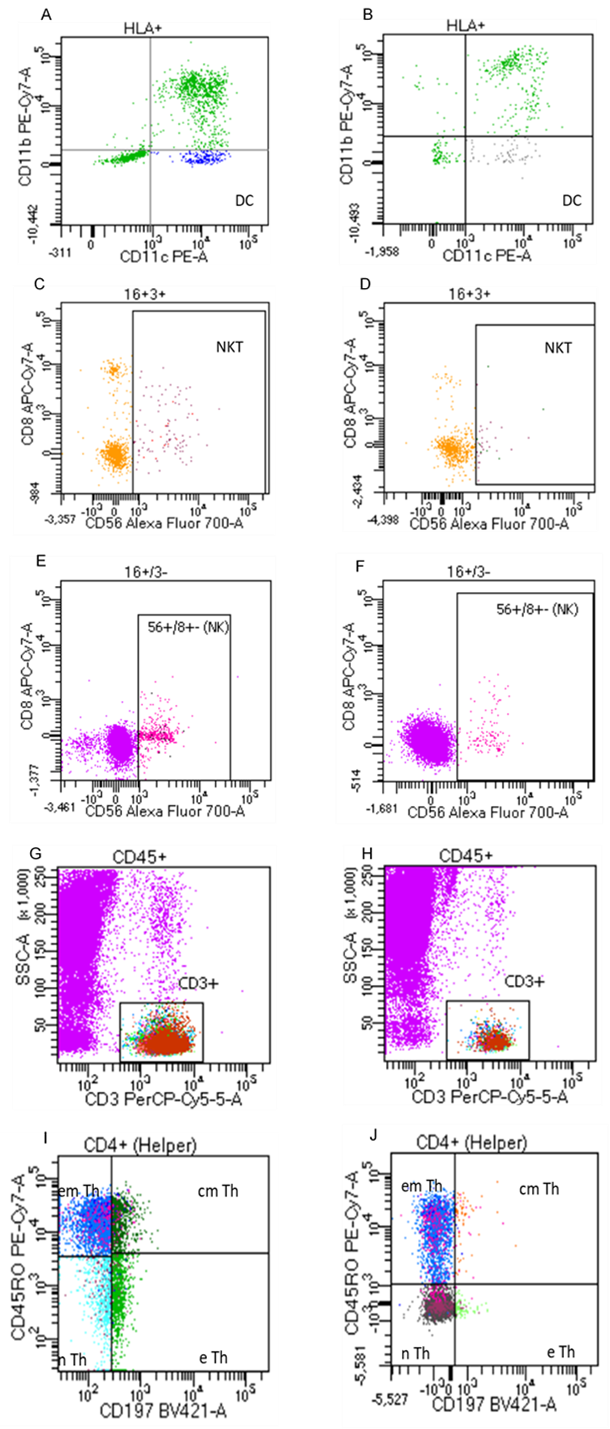


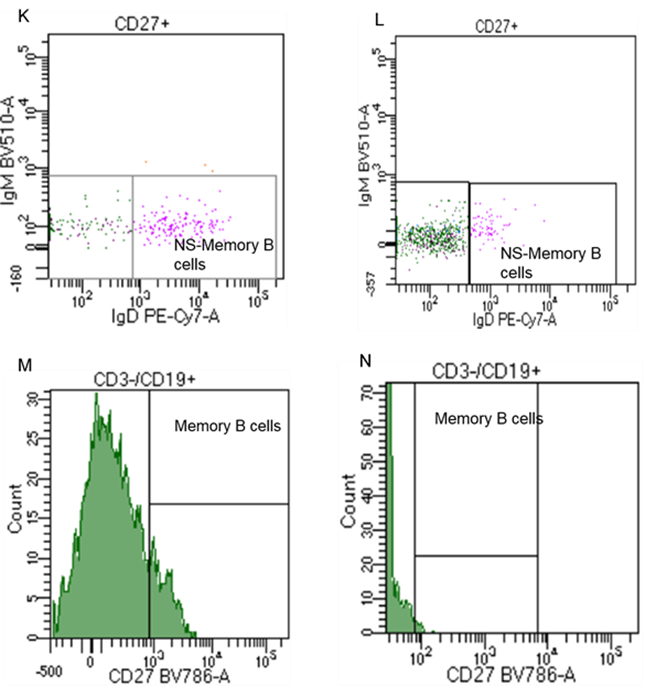


**Supplementary figure 5: Typical FCM pictures of DC, NKT, NK, T cells, subsets of Th, ns-memory B cells and Memory B cells in HD and HCC patients.** (A: DC in HD; B: DC in HCC; C: NKT in HD; D: NKT in HCC; E: NK in HD; F: NK in HCC; G: T cells in HD; H: T cells in HCC; I: Subsets of Th in HD; J: Subsets of Th in HCC; K: ns-memory B cells in HD; L: ns-memory B cellsin HCC; M: Memory B cells in HD; N: Memory B cells in HCC)


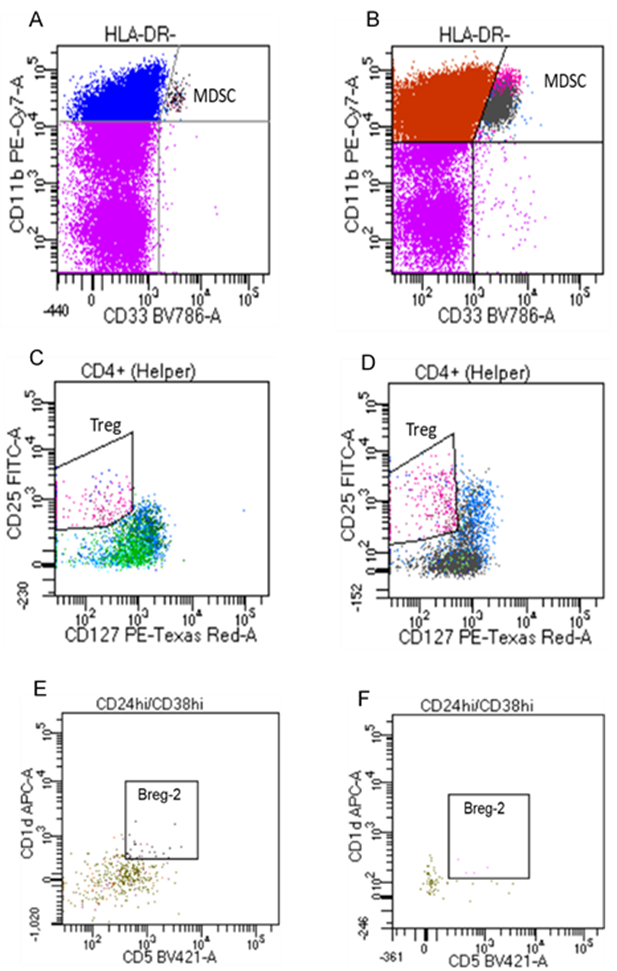


**Supplementary figure 6: Typical FCM pictures of MDSC, Treg and Breg-2 in HD and HCC patients.** (A: MDSC in HD; B: MDSC in HCC; C: Treg in HD; D: Treg in HCC; E: Breg-2 in HD; F: Breg-2 in HCC)


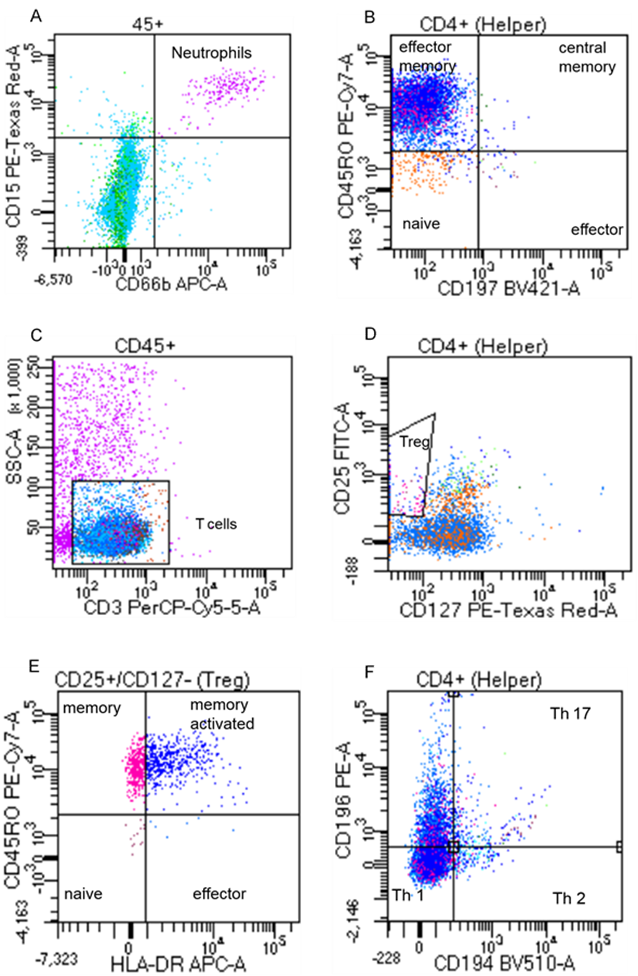


**Supplementary figure 7: Typical FCM pictures of immune cells in freshly harvested HCC tissues.**(A: Neutrophils; B: emTh,cmTh, eTh and nTh; C: T cells; D: Tregs; E: mTregs,maTregs,nTregs and aTregs; F: Th1; Th2 and Th17).


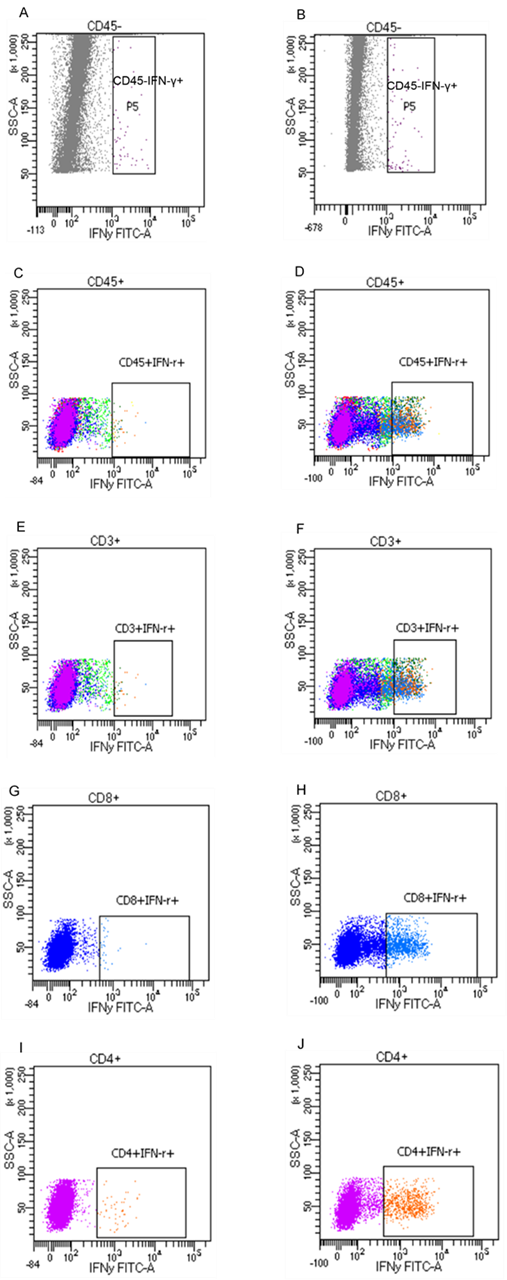


**Supplementary figure 8: Typical FCM pictures of IFN-γ in unstimulated group and stimulated group.** (A: IFN-γ of CD45^-^ cell populations in unstimulated group; B: IFN-γ of CD45^-^ cell populations in stimulated group; C: IFN-γ of CD45^+^ cells in unstimulated group; D: IFN-γ of CD45^+^ cells in stimulated group; E: IFN-γ of CD3^+^ cells in unstimulated group; F: IFN-γ of CD3^+^ cells in stimulated group; G: IFN-γ of CD8^+^ cells in unstimulated group; H: IFN-γ of CD8^+^ cells in stimulated group; I: IFN-γ of CD4^+^ cells in unstimulated group; J: IFN-γ of CD4^+^ cells in stimulated group)


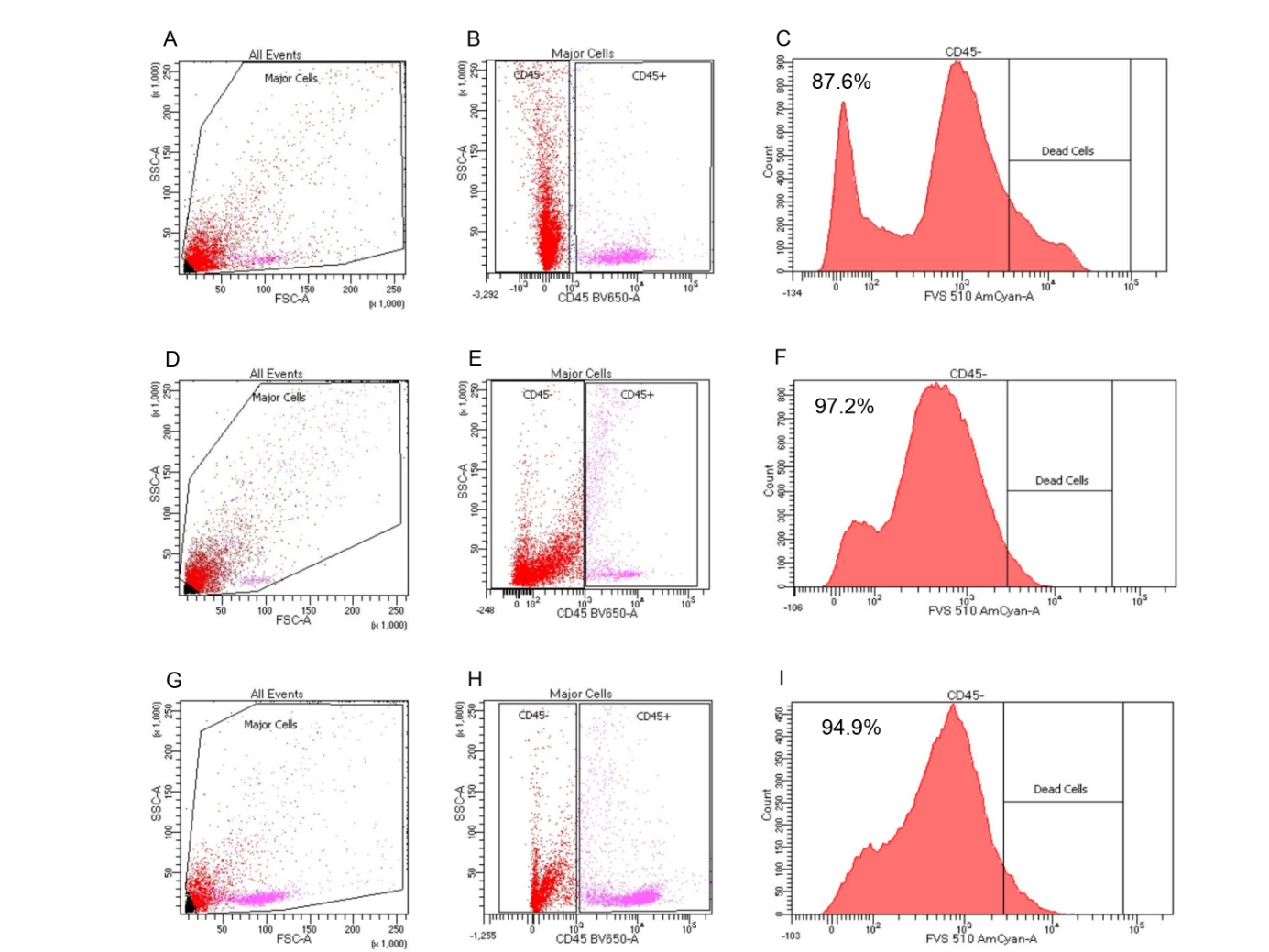


**Supplement Figure 9:** Typical FCM pictures of primary HCC cells at 0h and 24h (Co-culture of primary HCC cells with TILs, 1:10). (A)Major cells of HCC-0h; (B) CD45^-^ and C45^+^ cells in major cells at 0h; (C) Viability of HCC-0h; (D) Major cells of mono-HCC-24h; (E) CD45^-^ and C45^+^ cells in mono-HCC-24h; (F) Viability of mono-HCC-24h; (G) Major cells of co-HCC-24h; (H) CD45^-^ and CD45^+^ cells in co-HCC-24h; (I) Viability of co-HCC-24h.


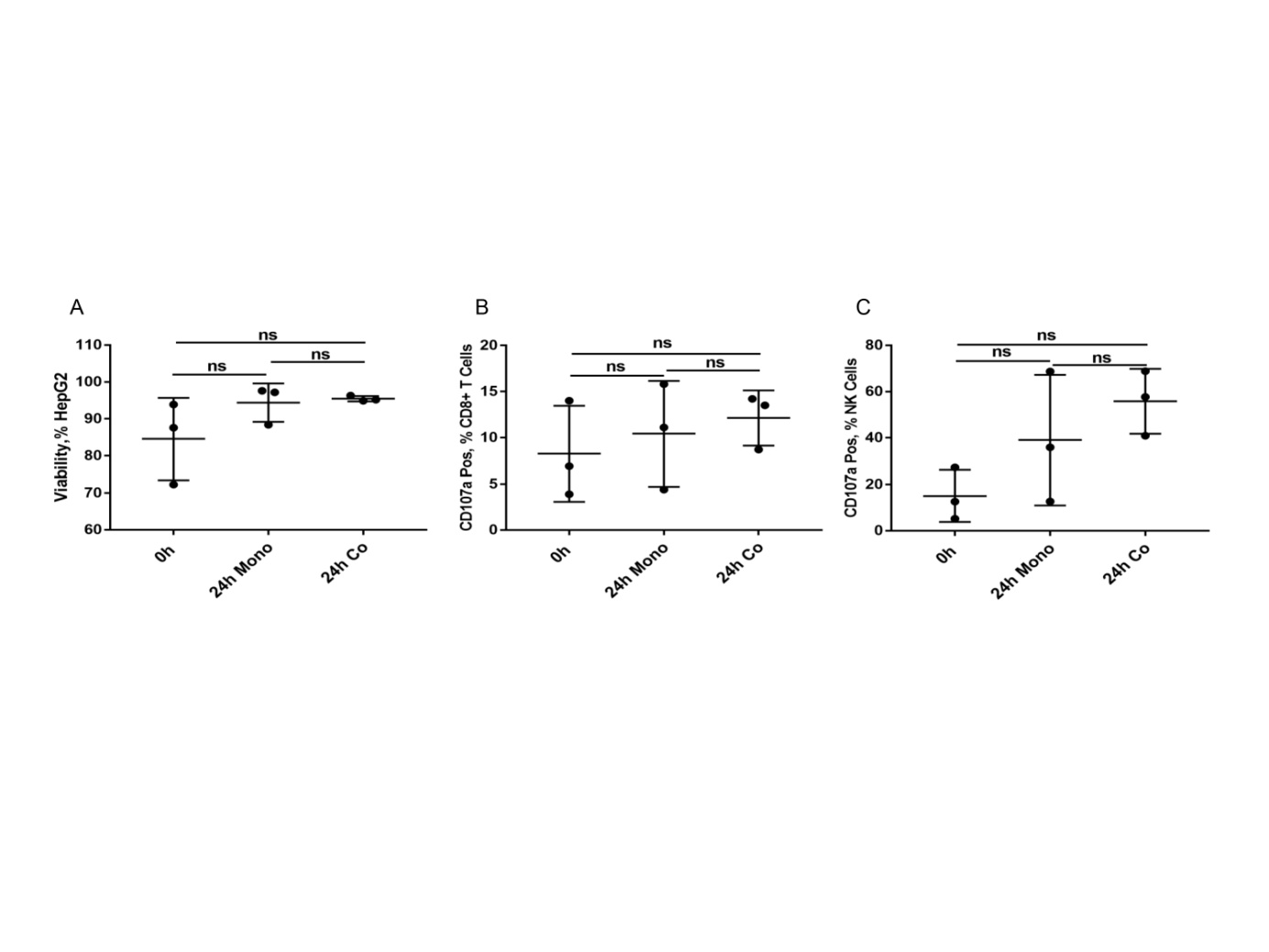


**Supplement Figure 10:** Statistical analysis of three repeated experiments (Co-culture of primary HCC cells with TILs, 1:10). (A) Viability of primary HCC cells; (B)Degranulation of CD8+ T lymphocytes; (C) Degranulation of NK cells. (One-Way ANOVA, ns: no significance)


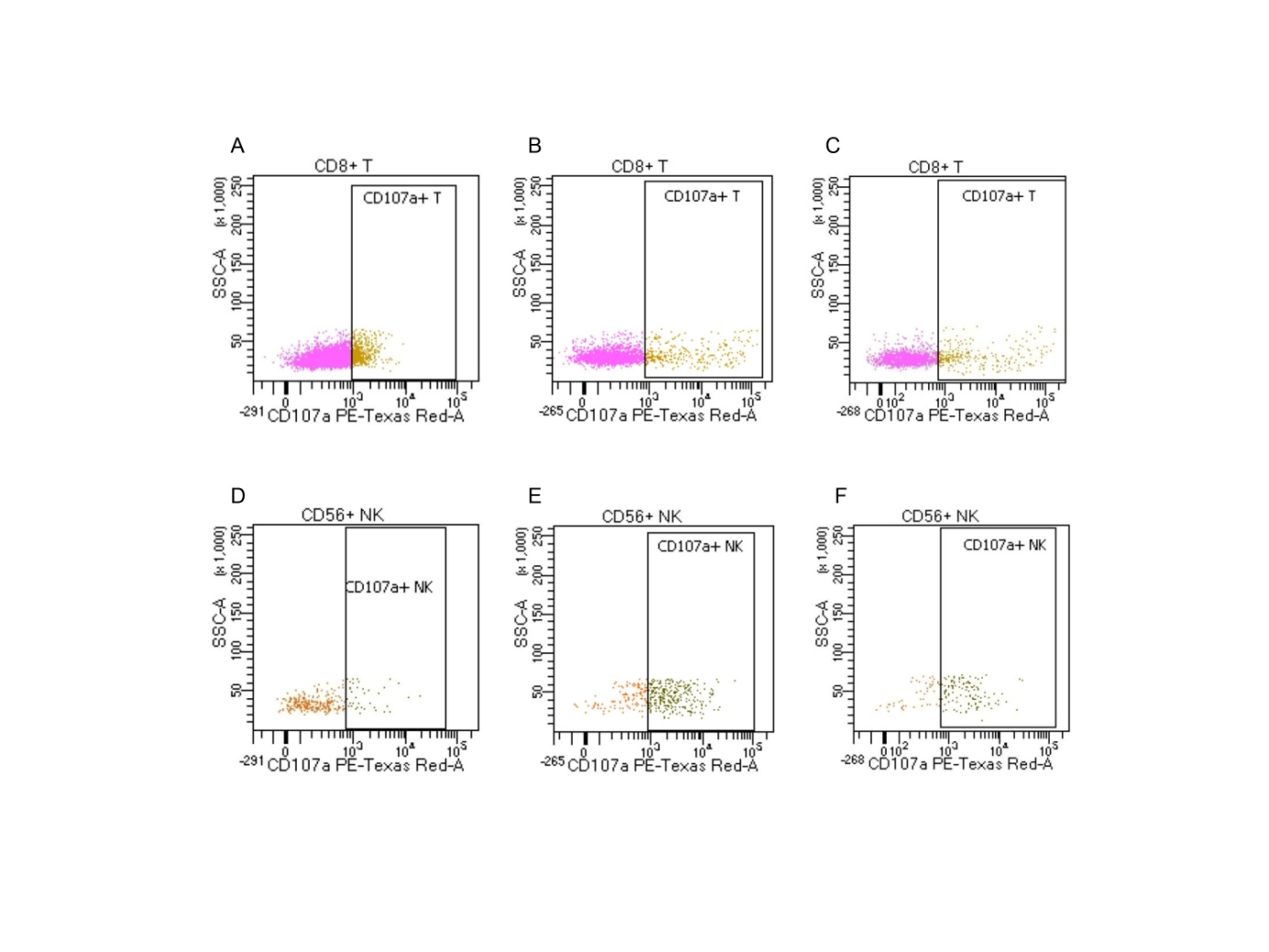


**Supplement Figure 11:** Typical FCM pictures of degranulation for CD8+ T lymphocytes and NK cells (Co-culture of primary HCC cells with TILs, 1:10). (A) Degranulation of CD8+ T-0h; (B) Degranulation of mono-CD8+ T-24h; (C)Degranulation of co-CD8+ T-24h; (D) Degranulation of NK-0h; (E) Degranulation of mono-NK-24h; (F) Degranulation of co-NK-24h.
