# Supplementary material for: Highly differential count of circulating and tumor infiltrating immune cells in patients with non-HCV/non-HBV hepatocellular carcinoma
Source: Cancer Immunol Immunother. 2021 Sep 28;71(5):1103–13. doi: 10.1007/s00262-021-03061-9 (PMC9015997; doi:10.1007/s00262-021-03061-9)
Supplement: Supplementary file 2 — Supplementary file2 (DOCX 35 KB) [file 262_2021_3061_MOESM2_ESM.docx]

#### Supplemental Table 1: Definition of measured cell subsets.

| **Cell type** | | **Marker** | |
| --- | --- | --- | --- |
| T cells, % of Leukocytes | | CD3^+^, % of CD45^+^ | |
| CD4^+^ T cells , % of T cells | | CD4^+^/CD8^-^, % of CD45^+^/CD3^+^ | |
| CD8^+^ T cells, % of T cells | | CD8^+^/CD4^-^, % of CD45^+^/CD3^+^ | |
| Th1, % of T cells | | CD4^+^/CD8^-^/CCR4^-^/CCR6^-^, % of CD45^+^/CD3^+^ | |
| Th2, % of T cells | | CD4^+^/CD8^-^/CCR4^+^/CCR6^-^, % of CD45^+^/CD3^+^ | |
| Th17, % of T cells | | CD4^+^/CD8^-^/CCR4^+^/CCR6^+^,% of CD45^+^/CD3^+^ | |
| Effector memory T cells, % of T cells | | CCR7^-^/CD45RO^+^, % of CD45^+^/CD3^+^ | |
| Central memory T cells, % of T cells | | CCR7^+^/CD45RO^+^, % ofCD45^+^/CD3^+^ | |
| Effector T cells, % of T cells | | CCR7^-^/CD45RO^-^, % of CD45^+^/CD3^+^ | |
| Naïve T cells, % of T cells | | CCR7^+^/CD45RO^-^, % of CD45^+^/CD3^+^ | |
| Activated T cells, % of T cells | | HLA_DR^+^/CD38^+^, % of CD45^+^/CD3^+^ | |
| Regulatory T-cells (T reg), % of T cells | | CD4^+^/CD8^-^/ CD25^+^/CD127^-^, % of CD45^+^/CD3^+^ | |
| Memory T reg, % of Treg | | HLA-­DR^-^/CD45RO^+^, % of CD45^+^/CD3^+^/CD4^+^/CD8^-^/ CD25^+^/CD127^-^ | |
| Naive T reg, % of Treg | | HLA-­DR^-^/CD45RO^-^, % of CD45^+^/CD3^+^CD4^+^/CD8^-^/ CD25^+^/CD127^-^ | |
| Activated T reg, % of Treg | | HLA­-DR^+^/CD45RO^-^, % of CD45^+^/CD3^+^ CD4^+^/CD8^-^/ CD25^+^/CD127^-^ | |
| Memory-Activated T reg, % of Treg | | HLA-DR^+^/CD45RO^+^, % of CD45^+^/CD3^+^ CD4^+^/CD8^-^/ CD25^+^/CD127^-^ | |
| B cells, % of Leukocytes | | | CD3^-^/CD19^+^, % of CD45^+^ |
| Memory B cells, % of B cells | | | CD27^+^, % of CD45^+^/CD19^+^/CD3^-^ |
| Class-switched Memory B cells, % of B cells | | | CD27^+^/IgD^-^/IgM^-^/CD20^+^/CD38^+^, % of CD45^+^/CD19^+^/CD3^-^ |
| Plasmablast, % of B cells | | | CD27^+^/IgD^-^/IgM^-^/CD20^-^/CD38^hi^, % of CD45^+^/CD19+/CD3- |
| Breg-1 B cells, % of B cells | | | CD27^+^/IgD^-^/IgM^-^/CD20^-^/CD38^hi^/CD5^+^, % of CD45^+^/CD19^+^/CD3^-^ |
| Non class-switched Memory B cells, % of B cells | | | CD27^+^/ IgD^+^, % of CD45^+^/CD19^+^/CD3^-^ |
| Naïve B cells, % of B cells | | | CD27^-^/IgD^+^, % of CD45^+^/CD19^+^/CD3^-^ |
| Transitional B cells, % of B cells | | | CD24^hi^/CD38^hi^, % of CD45^+^/CD19^+^/CD3^-^ |
| Breg-2 B cells, % of B cells | | | CD24^hi^/CD38^hi^/CD1d^+^/CD5^+^, % of CD45^+^/CD19^+^/CD3^-^ |
| Pro B cells, % of B cells | | | CD24^hi^/CD38^hi^/CD10^+^/IgM^-^, % of CD45^+^/CD19^+^/CD3^-^ |
| Pre B cells, % of B cells | | | CD24^hi^/CD38^hi^/CD10^+^/IgM^-^/CD20^+^, % of CD45^+^/CD19^+^/CD3^-^ |
| Plasma cells, % of B cells | | | CD10^-^/IgD^-^/IgM^-^/CD27^hi^/CD38^hi^, % of CD45^+^/CD19^+^/CD3^-^ |
| Neutrophils, % of Leukocytes | CD66b^+^/CD15^+^, % of CD45^+^ | | |
| Monocytes, % of Leukocytes | CD14^+^/CD33^+^, % of CD45^+^ | | |
| Macrophages, % of Leukocytes | CD33^+^/CD11b^+^/CD11c^+^/CD68^+^, % of CD45^+^ | | |
| Dendritic cell, % of Leukocytes | CD33^+^/HLA_DR^+^/CD11b^-^/CD11c^+^, % of CD45^+^ | | |
| MDSC, % of Leukocytes | HLA-DR^-^/ CD11b^+^/CD33^+^, % of CD45^+^ | | |
| G-MDSC, % of Leukocytes | HLA-DR^-^/ CD11b^+^/CD33^+^/CD14^-^/CD15^+^, % of CD45^+^ | | |
| M-MDSC, % of Leukocytes | HLA-DR^-^/ CD11b^+^/CD33^+^/CD14^+^/CD15^-^, % of CD45^+^ | | |
| NK cell, % of Leukocytes | CD3^-^/CD16^+^/CD56^+^/CD8^+-^, % of CD45^+^ | | |
| NKT cell, % of Leukocytes | CD3^+^/CD16^+^/CD56^+^/CD8^+-^, % of CD45^+^ | | |

#### Supplement table 2: Statistical calculations for all detected subsets between healthy donors and HCC patients

| **Cell type** | **HD** | **HCC** | **p** |
| --- | --- | --- | --- |
|  | **(Mean± SEM ,Number)** | **(Mean±SEM ,Number)** | **value** |
| Neutrophils, % of Leukocytes | 55.79±2.16, n=10 | 64.23±6.724, n=10 | 0.248 |
| Monocytes, % of Leukocytes | 5.412±0.561, n=10 | 4.363±0.745, n=10 | 0.275 |
| Macrophages, % of Leukocytes | 0.839±0.149, n=10 | 0.317±0.076, n=10 | 0.006 |
| Dendritic cell, % of Leukocytes | 0.387±0.077, n=10 | 0.124±0.045, n=10 | 0.009 |
| MDSC, % of Leukocytes | 0.564±0.139, n=10 | 2.005±0.64, n=10 | 0.041 |
| G-MDSC, % of MDSC | 40±7.408, n=10 | 28±8.142, n=10 | 0.290 |
| M-MDSC, % of MDSC | 35.26±7.754, n=10 | 46.9±9.318, n=10 | 0.350 |
| NK cell, % of Leukocytes | 3.42±0.52, n=10 | 1.943±0.588, n=10 | 0.076 |
| NKT cell, % of Leukocytes | 0.236±0.085, n=10 | 0.063±0.012, n=10 | <0.001 |
| B cells, % of Leukocytes | 2.611±0.258, n=10 | 1.901±0.47, n=10 | 0.202 |
| Non class-switched Memory B cells, %of B cells | 8.438±1.599, n=10 | 3.585±1.274, n=10 | 0.005 |
| Naïve B cells, % of B cells | 61.67±7.461, n=10 | 50.2±9.042, n=10 | 0.341 |
| Class-switched Memory B cells, % of B cells | 8.254±2.444, n=10 | 5.275±1.272, n=10 | 0.294 |
| Plasma cells, % of B cells | 4.464±1.525, n=10 | 2.061±0.861, n=10 | 0.187 |
| Plasmablast, % of B cells | 0.029±0.019, n=10 | 0.199±0.109, n=10 | 0.143 |
| Transitional B cells, % of B cells | 28.46±7.268, n=10 | 11.4±4.352, n=10 | 0.059 |
| Breg-2 B cells, % of B cells | 1.65±0.498, n=10 | 0.322±0.257, n=10 | 0.014 |
| Pro B cells, % of B cells | 7.346±2.685, n=10 | 2.061±0.746, n=10 | 0.074 |
| Pre B cells, % of B cells | 2.855±0.93, n=10 | 1.382±0.716, n=10 | 0.225 |
| Memory B cells, % of B cells | 31.57±8.962, n=10 | 11.6±2.697, n=10 | 0.047 |
| Breg-1 B cells, % of B cells | 0.001±0.001, n=10 | 0±0, n=10 | 0.331 |
| T cells, % of Leukocytes | 30.93±1.624, n=10 | 19.15±3.969, n=10 | 0.013 |
| CD4^+^ T cells , % of T cells | 67.76±2.105, n=10 | 66.75±5.762, n=10 | 0.872 |
| Th17, % of T cells | 15.68±3.109, n=10 | 18.07±3.536, n=10 | 0.618 |
| Th1, % of T cells | 54.3±5.199, n=10 | 48.61±5.398, n=10 | 0.458 |
| Th2, % of T cells | 13.08±2.439, n=10 | 13.14±2.59, n=10 | 0.988 |
| Effector memory CD4^+^ T cells, % of CD4^+^ T cells | 34.54±4.264, n=10 | 55.92±4.672, n=10 | 0.003 |
| Central memory CD4^+^ T cells, % of CD4^+^ T cells | 21.85±3.142, n=10 | 10.45±2.334, n=10 | 0.009 |
| Effector CD4^+^ T cells, % of CD4^+^ T cells | 16.62±4.722, n=10 | 24.28±5.388, n=10 | 0.299 |
| Naïve CD4^+^ T cells, % of CD4^+^ T cells | 26.99±4.221, n=10 | 9.354±3.041, n=10 | 0.003 |
| Activated CD4^+^ T cells, % of CD4^+^ T cells | 0.752±0.181, n=10 | 1.205±0.192, n=10 | 0.103 |
| CD8^+^ T cells, % of T cells | 25.59±2.15, n=10 | 27.83±5.393, n=10 | 0.704 |
| Effector memory CD8^+^ T cells, % of CD8^+^ T cells | 38.5±5.472, n=10 | 34.78±4.578, n=10 | 0.609 |
| Central memory CD8^+^ T cells, % of CD8^+^ T cells | 11.05±1.518, n=10 | 7.848±3.472, n=10 | 0.410 |
| Effector CD8^+^ T cells, % of CD8^+^ T cells | 33.4±4.314, n=10 | 42.9±8.707, n=10 | 0.341 |
| Naïve CD8^+^ T cells, % of CD8^+^ T cells | 17.06±4.462, n=10 | 14.46±5.763, n=10 | 0.726 |
| Activated CD8^+^ T cells, % of CD8^+^ T cells | 1.728±0.372, n=10 | 4.395±1.323, n=10 | 0.068 |
| Regulatory T-cells (Treg), % of T cells | 6.487±0.668, n=10 | 10.2±1.519, n=10 | 0.038 |
| Memory Treg, % of Treg | 59.36±2.075, n=10 | 64.45±3.667, n=10 | 0.242 |
| Memory-Activated Treg, % of Treg | 14.53±2.338, n=10 | 14.36±2.687, n=10 | 0.962 |
| Naive Treg, % of Treg | 25.82±1.55, n=10 | 20.98±3.82, n=10 | 0.255 |
| Activated Treg, % of Treg | 0.19±0.058, n=10 | 0.215±0.056, n=10 | 0.761 |
| CD4^+^ T cells/ CD8^+^ T cells | 2.918±0.377, n=10 | 3.911±1.048, n=10 | 0.384 |
| Th1/Th2 | 8.256±3.04, n=10 | 7.576±2.765, n=10 | 0.870 |
| Th1/Th17 | 6.476±2.375, n=10 | 4.704±1.567, n=10 | 0.541 |
| neutrophils/lymphocyte | 1.547±0.184, n=10 | 6.51±2.575, n=10 | 0.071 |

#### Supplement table 3: Statistical calculations for all detected subsets between peripheral blood and tumor tissues of HCC patients.

| **Cell type** | **Blood** | **Tumor** | **p** |
| --- | --- | --- | --- |
|  | **(Mean±SEM, Number)** | **(Mean± SEM, Number)** | **value** |
| Neutrophils, % of Leukocytes | 55.92±10.07, n=5 | 0.846±0.249, n=5 | 0.005 |
| Monocytes, % of Leukocytes | 5.124±1.183, n=5 | 1.974±0.619, n=5 | 0.028 |
| Macrophages, % of Leukocytes | 0.414±0.132, n=5 | 0.314±0.082, n=5 | 0.265 |
| Dendritic cell, % of Leukocytes | 0.16±0.086, n=5 | 0.142±0.041, n=5 | 0.837 |
| MDSC, % of Leukocytes | 2.376±1.262, n=5 | 1.834±0.869, n=5 | 0.763 |
| G-MDSC, % of MDSC | 0.352±0.100, n=5 | 1.678±0.754, n=5 | 0.077 |
| M-MDSC, % of MDSC | 1.326±0.892, n=5 | 4.464±2.67, n=5 | 0.028 |
| NK cell, % of Leukocytes | 2.298±1.013, n=5 | 2.306±1.166, n=5 | 0.993 |
| NKT cell, % of Leukocytes | 0.06±0.012, n=5 | 8.132±5.152, n=5 | 0.192 |
| B cells, % of Leukocytes | 2.496±0.712, n=5 | 2.308±1.816, n=5 | 0.868 |
| Non class-switched Memory B cells, %of B cells | 2.938±0.956, n=5 | 1.008±1.013, n=5 | 0.257 |
| Naïve B cells, % of B cells | 51.41±13.03, n=5 | 57.83±18.34, n=5 | 0.733 |
| Class-switched Memory B cells, % of B cells | 6.326±2.3, n=5 | 1.314±1.388, n=5 | 0.145 |
| Plasma cells, % of B cells | 1.58±0.813, n=5 | 0.152±0.304, n=5 | 0.182 |
| Plasmablast, % of B cells | 0.304±0.214, n=5 | 0±0, n=5 | 0.228 |
| Transitional B cells, % of B cells | 14.03±7.878, n=5 | 6.596±6.327, n=5 | 0.485 |
| Breg-2 B cells, % of B cells | 0.58±0.513, n=5 | 0.376±0.31, n=5 | 0.686 |
| Pro B cells, % of B cells | 2.73±1.303, n=5 | 0.462±0.749, n=5 | 0.204 |
| Pre B cells, % of B cells | 2.536±1.274, n=5 | 0.462±0.749, n=5 | 0.235 |
| Memory B cells, % of B cells | 12.72±3.492, n=5 | 5.584±1.93, n=5 | 0.162 |
| Breg-1 B cells, % of B cells | 0±0, n=5 | 0±0, n=5 |  |
| T cells, % of Leukocytes | 22.84±5.435, n=5 | 58.17±7.135, n=5 | 0.021 |
| CD4^+^ T cells , % of T cells | 73.82±4.664, n=5 | 42.61±6.704, n=5 | 0.001 |
| Th17, % of T cells | 19.89±6.429, n=5 | 3.402±2.097, n=5 | 0.057 |
| Th1, % of T cells | 48.23±9.415, n=5 | 77±9.883, n=5 | 0.042 |
| Th2, % of T cells | 12.03±2.847, n=5 | 3.538±0.318, n=5 | 0.046 |
| Effector memory CD4^+^ T cells, % of CD4^+^ T cells | 57.28±7.237, n=5 | 78.43±4.772, n=5 | 0.042 |
| Central memory CD4^+^ T cells, % of CD4^+^ T cells | 8.406±2.154, n=5 | 0.09±0.053, n=5 | 0.018 |
| Effector CD4^+^ T cells, % of CD4^+^ T cells | 28.13±10.09, n=5 | 21.15±4.916, n=5 | 0.473 |
| Naïve CD4^+^ T cells, % of CD4^+^ T cells | 6.182±2.197, n=5 | 0.33±0.203, n=5 | 0.060 |
| Activated CD4^+^ T cells, % of CD4^+^ T cells | 1.052±0.214, n=5 | 2.706±1.559, n=5 | 0.380 |
| CD8^+^ T cells, % of T cells | 20.35±3.405, n=5 | 21.18±2.253, n=5 | 0.742 |
| Effector memory CD8^+^ T cells, % of CD8^+^ T cells | 38.9±5.715, n=5 | 38.24±9.823, n=5 | 0.951 |
| Central memory CD8^+^ T cells, % of CD8^+^ T cells | 11.33±6.671, n=5 | 0.052±0.024, n=5 | 0.167 |
| Effector CD8^+^ T cells, % of CD8^+^ T cells | 43.82±10.81, n=5 | 61.62±9.766, n=5 | 0.164 |
| Naïve CD8^+^ T cells, % of CD8^+^ T cells | 5.954±2.11, n=5 | 0.088±0.05, n=5 | 0.052 |
| Activated CD8^+^ T cells, % of CD8^+^ T cells | 4.192±1.777, n=5 | 3.436±3.201, n=5 | 0.873 |
| Regulatory T-cells (Treg), % of T cells | 9.062±2.335, n=5 | 7.022±3.991, n=5 | 0.694 |
| Memory Treg, % of Treg | 62.53±4.788, n=5 | 33.25±8.986, n=5 | 0.018 |
| Memory-Activated Treg, % of Treg | 16.08±4.108, n=5 | 18.74±9.716, n=5 | 0.731 |
| Naive Treg, % of Treg | 21.17±5.782, n=5 | 23.89±12.41, n=5 | 0.805 |
| Activated Treg, % of Treg | 0.214±0.037, n=5 | 4.116±2.743, n=5 | 0.228 |
| CD4^+^ T cells/ CD8^+^ T cells | 4.26±0.969, n=5 | 2.188±0.508, n=5 | 0.024 |
| Th1/Th2 | 8.182±4.91, n=5 | 21.54±1.522, n=5 | 0.038 |
| Th1/Th17 | 5.274±3.002, n=5 | 139±53.86, n=5 | 0.064 |
| neutrophils/lymphocyte | 2.964±1.128, n=5 | 0.01±0.003, n=5 | 0.059 |
